# Endothelial *Rbpj* deletion normalizes Notch4-induced brain arteriovenous malformation in mice

**DOI:** 10.1084/jem.20211390

**Published:** 2022-11-28

**Authors:** Corinne M. Nielsen, Xuetao Zhang, Kunal Raygor, Shaoxun Wang, Andrew W. Bollen, Rong A. Wang

**Affiliations:** 1 Laboratory for Accelerated Vascular Research, Department of Surgery, University of California, San Francisco, San Francisco, CA; 2 Department of Pathology, University of California, San Francisco, San Francisco, CA

## Abstract

Upregulation of Notch signaling is associated with brain arteriovenous malformation (bAVM), a disease that lacks pharmacological treatments. Tetracycline (tet)-regulatable endothelial expression of constitutively active Notch4 (Notch4*^tetEC^) from birth induced bAVMs in 100% of mice by P16. To test whether targeting downstream signaling, while sustaining the causal Notch4*^tetEC^ expression, induces AVM normalization, we deleted *Rbpj*, a mediator of Notch signaling, in endothelium from P16, by combining tet-repressible Notch4*^tetEC^ with tamoxifen-inducible *Rbpj* deletion. Established pathologies, including AV connection diameter, AV shunting, vessel tortuosity, intracerebral hemorrhage, tissue hypoxia, life expectancy, and arterial marker expression were improved, compared with Notch4*^tetEC^ mice without *Rbpj* deletion. Similarly, *Rbpj* deletion from P21 induced advanced bAVM regression. After complete AVM normalization induced by repression of Notch4*^tetEC^, virtually no bAVM relapsed, despite Notch4*^tetEC^ re-expression in adults. Thus, inhibition of endothelial *Rbpj* halted Notch4*^tetEC^ bAVM progression, normalized bAVM abnormalities, and restored microcirculation, providing proof of concept for targeting a downstream mediator to treat AVM pathologies despite a sustained causal molecular lesion.

## Teaser

Nielsen and Zhang et al. show that well-established, Notch4-induced brain arteriovenous malformations are normalized, following deletion of the Notch signaling mediator, *Rbpj*. Upon complete regression, virtually no AVM relapses in adults, even when the causal factor is reintroduced.

## Graphical Abstract

**Figure:**
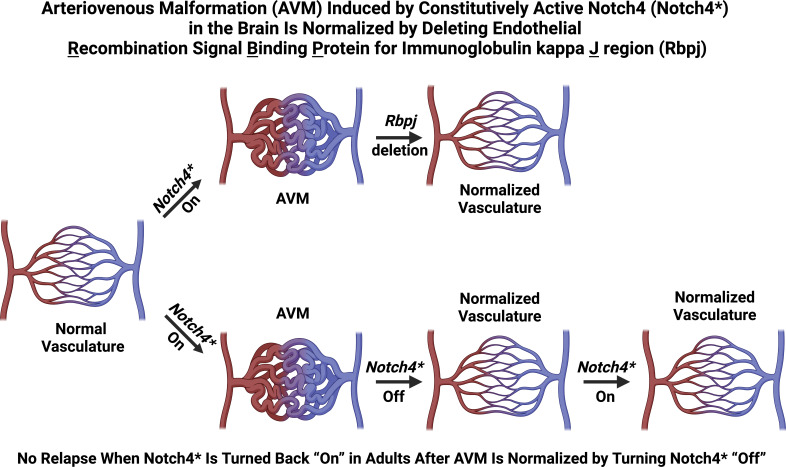

## Introduction

Brain arteriovenous (AV) malformation (bAVM) is a devastating neurovascular disease characterized by direct shunting (Hartmann et al., 1998), leading to tortuous, high-flow vessels tangled into a nidus, connecting feeding arteries directly to draining veins (Nielsen et al., 2014). bAVMs are prone to rupture, resulting in ischemia, hemorrhage, and neurological impairment (Hartmann et al., 1998; Rutledge et al., 2014). Treatment includes management of symptoms and/or invasive treatments to impede blood flow and/or surgical resection (Bendok et al., 2014; Chen et al., 2014; Friedlander, 2007; Lobov et al., 2007; Ponce and Spetzler, 2011; Rangel-Castilla et al., 2014; Zuurbier and Al-Shahi Salman, 2019). There is urgent need to understand mechanisms of disease pathogenesis and to develop noninvasive, molecular therapies against bAVMs.

Both inherited germline mutations and de novo somatic mutations may trigger bAVMs. Germline mutations leading to bAVM are rare and typically part of multisymptom diseases (Bayrak-Toydemir et al., 2006; Benzinou et al., 2012; Burger et al., 2002; Cole et al., 2005; Gallione et al., 2004; Johnson et al., 1996; Letteboer et al., 2015; McAllister et al., 1994; Olivieri et al., 2002; Scimone et al., 2020; Wooderchak-Donahue et al., 2013). Somatic activating mutations in *KRAS* (*KRAS*^*G12V*^) were recently identified, with high prevalence in bAVM patient samples (Nikolaev et al., 2018; Hong et al., 2019).

These findings have invigorated the field to seek other causal, somatic mutations and to determine crosstalk between KRAS/MAPK/ERK signaling and other pathways associated with bAVM. bAVMs often present as sporadic, focal lesions whose underlying causal mutation(s) remain unknown. Recent studies have uncovered genomic variances in *FLT4*, *CCN1*, *NOTCH4* (Delev et al., 2017; Scimone et al., 2020), *ANRIL*, *CDKN2A/B*, *miR-18a* (Florian et al., 2020), *Smad6* (Fu et al., 2020), *KLF4*, *SNAI1*, and *SNAI2* (Shoemaker et al., 2020), which may be associated with bAVM susceptibility. Expression studies in human bAVM tissue showed altered expressions of signaling molecules, including those in Notch signaling—DLL4, NOTCH1, and NOTCH4 (Davis et al., 2018; Hill-Felberg et al., 2015; Murphy et al., 2009; Sasahara et al., 2007; Thomas et al., 2018; ZhuGe et al., 2009)—suggesting that unidentified disruptions to cellular signaling pathways may contribute to AVM pathology. Continued studies may link these molecular lesions to *KRAS* mutations and promote the design of molecular-based bAVM therapies.

Notch signaling plays critical roles in vascular development, including AV specification (Fang et al., 2017; You et al., 2005) and sprouting angiogenesis (Benedito et al., 2009; Hellstrom et al., 2007; Lobov et al., 2007; Pitulescu et al., 2017; Suchting et al., 2007). Endothelial Notch signaling requires activation of the downstream transcription factor, namely recombination signal binding protein for immunoglobulin κ J region (Rbpj) to promote transcription of target genes (Bray, 2016; Giaimo and Borggrefe, 2018). Genetic studies in mice have revealed requirements for Notch signaling during embryonic vascular development and postnatal vascular maintenance (Cuervo et al., 2016; Kerr et al., 2016; Krebs et al., 2004; Krebs et al., 2010; Krebs et al., 2000; Nielsen et al., 2014). Thus, Notch contributes to vascular homeostasis throughout mammalian life.

Inducible expression of constitutively active *Notch4*, in postnatal endothelium, termed Notch4*^tetEC^ transgene herein, leads to AVM and moribundity by postnatal day (P) 36 in mice (Murphy et al., 2008). Notch4*^tetEC^ is controlled by the tetracycline (tet)-repressible system, such that administration of tet represses Notch4*^tetEC^ expression. Turning off the causal Notch4*^tetEC^ transgene elicits bAVM regression (Murphy et al., 2012) and extends survival (Murphy et al., 2008) in mice, normalizing the established AVM to microvessels. Here, we tested a new treatment strategy that inhibits downstream Notch signaling without switching off the causal Notch4*^tetEC^ transgene. We deleted *Rbpj*, a mediator of Notch, and showed regression of established bAVMs in Notch4*^tetEC^ mice.

## Results and discussion

### Expression of Notch4*^tetEC^ from birth led to well-established AVM with minimal animal illness by P16

As AVM pathogenesis was well established in 100% of P16 *Tie-tTA*;TRE-Notch4* (Notch4*^tetEC^) brains, in which tet was withdrawn from birth and thus Notch4*^tetEC^ was induced from birth, we chose P16 as the timepoint to induce AVM regression. Another advantage of P16 is that fewer mice had reached moribundity by this time, thus offering an opportunity to achieve AVM rescue in “healthier” mice, with fewer confounding effects of general illness. Furthermore, we considered the time it takes to achieve effective inhibition of Notch4* signaling and to induce AVM regression, following tamoxifen (TAM) injection at P16 and subsequent Rbpj deletion. After TAM administration, it takes time for Cre to be expressed and to excise the floxed *Rbpj* sequence, time for the cells to stop producing Rbpj, and time for the already-produced Rbpj to be degraded and cleared from cells, before Rbpj deletion is effectively achieved. Similar to how Rbpj is cleared from the cells, it also takes time to stop transmission of Notch4*^tetEC^ signaling and for the downstream targets to be cleared, and to achieve effective inhibition of Notch4*^tetEC^ signaling. Thus, the actual timing to abrogate Notch4*^tetEC^ signaling is after P16, when the Notch4*^tetEC^ mice had progressed into more mature AVM, and when more Notch4*^tetEC^ mice began to reach moribundity from ∼P18 (Murphy et al., 2008). Thus, TAM administration from P16 is an optimal time point to induce the regression of well-established AVMs.

**Figure S1.**
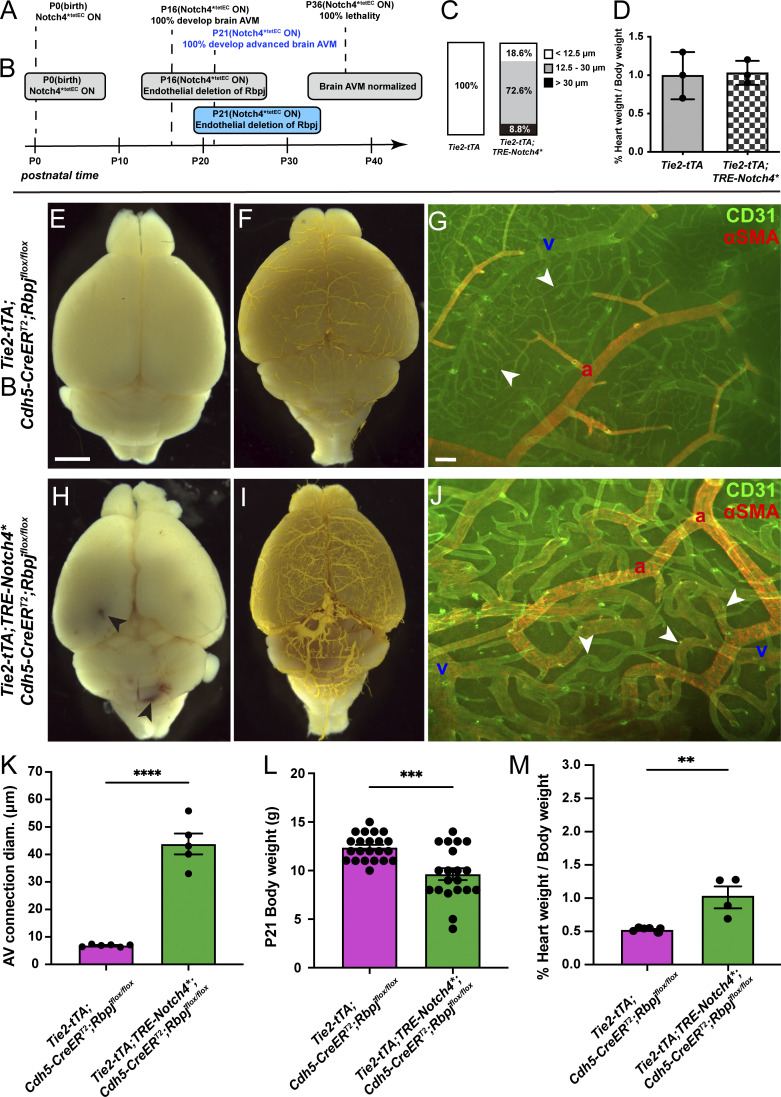
**Additional characterizations of brain AVM established by P16 and P21. (A and B)** Postnatal time is depicted along the timeline. **(A)** Constitutive activation of Notch4*^tetEC^ from birth led to features of brain AVM by P16 and to moribundity/death by P36. **(B)** Endothelial deletion of *Rbpj* from P16 attenuated features of Notch4*^tetEC^ induced brain AVM and increased the time to moribundity/death. **(C)** Normal AV connections were defined as <12.5 μm diameter (white box); moderately enlarged AV connections were defined as 12.5–30 μm diameter (gray box); severely enlarged AV connection were defined as >30 μm diameter (black box). In P16 control brains, 100% of AV connections were of normal diameter (*N* = 3 mice, 88 connections). In P16 Notch4*^tetEC^ brains, 72.6 and 8.8% of AV connection diameters were moderately and severely enlarged, respectively (*N* = 4 mice, 113 connections). Two independently repeated experiments. **(D)** Percentage of heart weight/total body weight was calculated for control (1.00 ± 0.3%, *N* = 3 mice) and Notch4*^tetEC^ (1.03 ± 0.15%, *N* = 3 mice) mice at P16, following Notch4*^tetEC^ activation from birth. No significant difference was seen among cohorts, suggesting that cardiomegaly did not develop by P16. P = 0.8796. Two independently repeated experiments. **(E and F)** *Tie2-tTA* perfused whole brain did not show evidence for cerebrovascular hemorrhages (*N* = 5) or vessel tortuosities (*N* = 3) at P21. **(G)** Flat-mount immunostaining against endothelial marker CD31 and smooth muscle cell marker αSMA showed normal αSMA expression restricted to arteries and absent from AV connections (arrowheads; *N* = 5). **(H and I)** Hemorrhages (arrowheads; *N* = 6) and enlarged and tortuous vessels (*N* = 3) were present in perfused *Tie2-tTA*;TRE-Notch4* brains at P21. **(J)** CD31 and αSMA immunostaining showed AV shunting (arrowheads) and veins with αSMA expression in P21 cortex from *Tie2-tTA*;TRE-Notch4* brains (*N* = 6). **(K)** Average diameter of AV connections was higher in *Tie2-tTA*;TRE-Notch4* mice (*N* = 6) at P21 as compared to *Tie2-tTA* negative controls (*N* = 5). P < 0.0001. **(L)** Body weight was decreased in *Tie2-tTA*;TRE-Notch4* mice (*N* = 20) at P21, as compared to *Tie2-tTA* controls (*N* = 22). P = 0.0002. **(M)** Percent of heart weight/body weight was higher in *Tie2-tTA*;TRE-Notch4* mice (*N* = 4) at P21, as compared to *Tie2-tTA* negative controls (*N* = 6). P = 0.0045. a, artery; v, vein. E–J, K, and M from four independently repeated experiments and L from 14 independently repeated experiments. Scale bars: 2 mm in E, F, H, and I, 100 μm in G and J. **P<0.01; ***P<0.001; ****P<0.0001.

**Figure 1.**
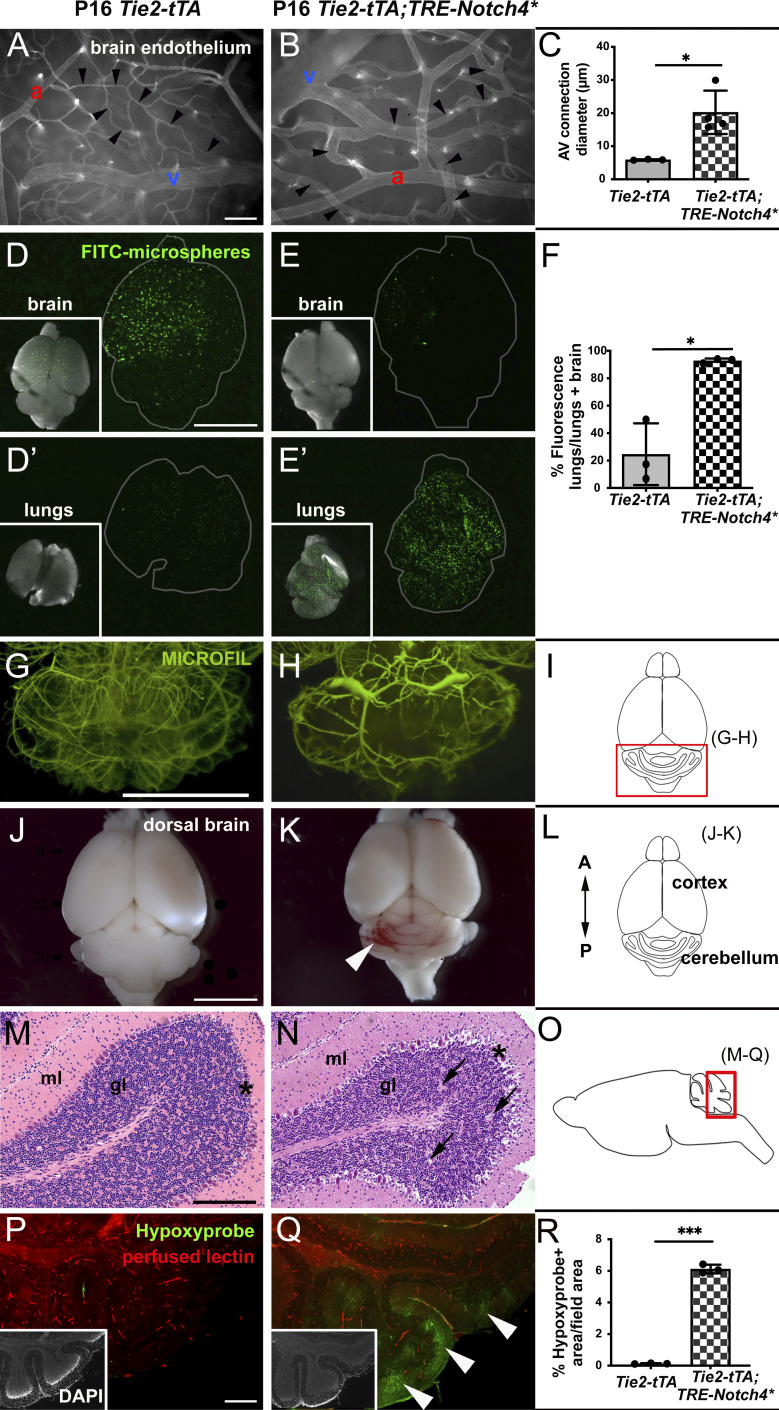
Expression of Notch4*^tetEC^ **from birth led to brain AVMs by P16. (A–C)** Whole-mount frontal cortex from P16 brain with FITC-lectin+ ECs to highlight blood vessels. Arrowheads indicate AV connections with capillary diameter in P16 control brains (A and C) and enlargement in Notch4*^tetEC^ brains (B and C). a, artery; v, vein. *N* = 4 Notch4*^tetEC^ mice (113 connections; 20.26 ± 3.28 μm); *N* = 3 controls (88 connections; 5.90 ± 0.08 μm). P = 0.0220. Two independently repeated experiments. **(D–F)** Microsphere passage assay. FITC-microspheres (15 μm diameter, too large to pass capillaries) were injected into the left common carotid artery to circulate. Microspheres lodged in capillaries in control P16 brains (D and D′); microspheres passed through AV shunts in Notch4*^tetEC^ P16 brains and lodged in lungs (E and E′). *N* = 3 Notch4*^tetEC^ mice (92.76 ± 1.64%); *N* = 3 controls (24.62 ± 22.47%). P = 0.0346. Three independently repeated experiments. **(G and H)** MICROFIL casting of P16 brain vasculature. Cerebellum is shown, as cartooned in **(I)**. Vessels were enlarged and tortuous in Notch4*^tetEC^ cerebellum (*N* = 3), as compared to the control (*N* = 3). Three independently repeated experiments. **(J and K)** Saline-perfused whole brain showed evidence for hemorrhage in Notch4*^tetEC^ brain (arrowhead) and not in control. Three independently repeated experiments. **(L)** Brain regions depicted in cartoon. A, anterior; P, posterior. *N* = 3 Notch4*^tetEC^ mice; *N* = 3 controls. **(M)** H&E staining showed normal histology in the sagittal section through P16 control cerebellum (*N* = 3). **(N)** Minor tissue lesions (arrows) and disrupted Purkinje neurons (asterisks) were seen in P16 Notch4*^tetEC^ cerebellum (*N* = 4). ml, molecular layer; gl, internal granule layer. Scale bar, 200 μm. Two independently repeated experiments. **(O)** Schematic indicates the cerebellar region of the sagittal brain section shown in M and N, P and Q. **(P and Q)** Hypoxyprobe immunostaining showed regions of hypoxic cells (arrowheads) in Notch4*^tetEC^ cerebellum but not in controls. Quantified in **(R)**. Lectin-positive staining indicated perfused vessels. *N* = 3 Notch4*^tetEC^ mice (10 fields; 6.12 ± 0.28%); *N* = 3 controls (12 fields; 0.13 ± 0.03%). P = 0.0007. Insets show DAPI+ nuclei. Three independently repeated experiments. Scale bars: 100 μm in A, B, P, and Q; 5 mm in D–E′, G, H, J, and K; 200 μm in M and N. *P<0.05; ***P<0.001.

We thus documented AVM pathogenesis at P16 as a reference point for AVM regression (Fig. S1, A and B). In all Notch4*^tetEC^ mice examined, mean brain AV diameter and the proportion of AV connections with diameter ≥12.5 μm were increased in Notch4*^tetEC^ mice (Fig. 1, A–C, and Fig. S1 C). To test for functional AV shunting, we performed a microsphere passage assay and found FITC-microspheres confined to brain capillaries in controls but lodged in lungs in Notch4*^tetEC^ mutants, indicating microsphere circulation through AV shunts by P16 (Fig. 1, D and E′).

Other features of bAVM developed by P16 in mice with Notch4*^tetEC^ expressed from birth*.* Morphologically, casts of brain vessels appeared more tortuous in Notch4*^tetEC^ vs. controls (Fig. 1, G–I), with AVM niduses emerging in Notch4*^tetEC^ brains at P16. Perfused Notch4*^tetEC^ brains, but not controls, showed evidence for hemorrhage by P16 (Fig. 1, J–L). Histological analysis revealed minor tissue lesions in Notch4*^tetEC^ brains by P16 (Fig. 1, M–O). Finally, we detected hypoxic cells in brain parenchyma in Notch4*^tetEC^ mice by P16, but not in controls (Fig. 1, P–R). We consistently observed bleeding and tortuous vessels in the cerebellum. Similarly, mice with mutations in genes responsible for cerebral cavernous malformations also develop cerebral cavernous malformations in mouse cerebellum (Zhou et al., 2016). The cerebellum is a region that undergoes extensive morphogenesis in immature brains (Acker et al., 2001; Chapman et al., 2022), suggesting that cerebellar endothelial cells (ECs) may be regionally and temporally susceptible to Notch4*^tetEC^ activation.

To accommodate increased blood flow through AV shunts and related systemic hemodynamic changes, the heart must compensate by increasing cardiac output and may develop compensatory cardiomegaly or ventricular hypertrophy (Carlson et al., 2005). Despite developing hallmarks of AVM by P16, Notch4*^tetEC^ mice did not display cardiomegaly or increased heart weight/body weight percentage (Fig. S1 D). Thus, while we observed several features of bAVM by P16, these likely did not alter systemic hemodynamics enough to lead to compensatory cardiomegaly. Together, our data demonstrate P16 as a time point at which all Notch4*^tetEC^ mice established bAVM pathologies.

### Expression of Notch4*^tetEC^ from birth led to severe AVM and compromised health by P21

We next established an experimental time point at P21 when Notch4*^tetEC^ AVM pathologies were more severe. As ∼75% Notch4*^tetEC^ mice reach moribundity by P21 (Murphy et al., 2008), and because surviving Notch4*^tetEC^ mice reach moribundity shortly after P21, we chose this timepoint to study the regression from severe AVMs. Gross morphological analysis and vascular casting revealed evidence of hemorrhage and vessel tortuosity (particularly in the cerebellum) in P21 Notch4*^tetEC^ brain but not controls (Fig. S1, E, F, H, and I). Immunostaining against CD31 and α-smooth muscle actin (αSMA) showed greatly enlarged and tortuous AV connections, with increased expression of the arterial marker αSMA in Notch4*^tetEC^ brains, as compared with controls (Fig. S1, G and J). AV connection diameters were significantly increased in P21 Notch4*^tetEC^ brain as compared with controls (Fig. S1 K). The mean body weight of Notch4*^tetEC^ mice was less than the negative controls (Fig. S1 L), and the percentage of heart weight/body weight of Notch4*^tetEC^ mice was greater than the negative controls (Fig. S1 M). These data suggest that expression of Notch4*^tetEC^ from birth led to severe brain AVM, with compromised health and systemic hemodynamic effects, by P21.

### Endothelial deletion of *Rbpj* from P16 delayed moribundity in Notch4*^tetEC^ mice

**Figure 2.**
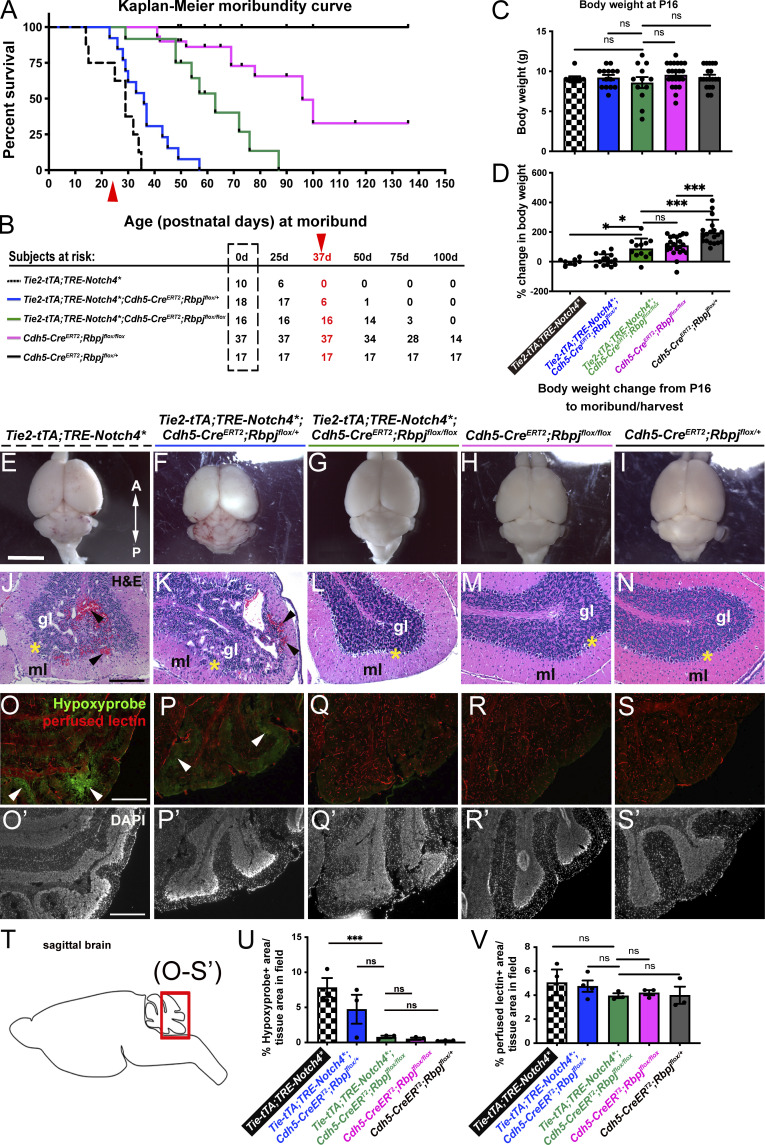
Endothelial deletion of *Rbpj* from P16 reduced the time to moribundity, body weight loss, intracerebral hemorrhage, histopathological abnormalities, and tissue hypoxia in Notch4*^tetEC^ **mice. (A)** Kaplan–Meier analysis showed that time to moribundity doubled in Notch4*^tetEC^;*Rbpj*^*iΔEC*^ mice (green line), as compared with Notch4*^tetEC^ mice (dashed black line). P < 0.0001. Red arrowhead indicates age (P36) past which no Notch4*^tetEC^ mice are expected to survive. Notch4*^tetEC^;*Rbpj*^*iΔEC-het*^ mice (blue line) did not increase time to moribundity, as compared to Notch4*^tetEC^ mice. *Rbpj*^*iΔEC*^ mice (pink line) increased time to moribundity but did not match the curve of negative control mice (solid black line). 13 independently repeated experiments. **(B)** Numbers of subjects at risk at 0, 25, 50, 75, 100 d old were used to generate Kaplan–Meier curve. Red arrowhead indicates age (P36) past which no Notch4*^tetEC^ mice are expected to survive. **(C)** At P16, the age of initial TAM injection to induce *Rbpj*^*iΔE*C^ deletion, body weights of mice in all genotypic cohorts were not significantly different from one another. **(D)** Changes in body weights, from P16 to time of moribundity/tissue harvest, were calculated. Notch4*^tetEC^;*Rbpj*^*iΔEC*^ mice (green bar, *N* = 12; 86.90 ± 78.87%) gained significantly more weight than Notch4*^tetEC^ mice (checkered bar, *N* = 8; −0.49 ± 20.71%) and Notch4*^tetEC^;*Rbpj*^*iΔEC-het*^ mice (blue bar, *N* = 14; 17.18 ± 39.19%); however, Notch4*^tetEC^;*Rbpj*^*iΔEC*^ mice (green bar) did not reach weight gain of negative control mice (dark gray bar, *N* = 20; 159.83 ± 45.18%). Notably, *Rbpj*^*iΔEC*^ mice (pink bar, *N* = 23; 99.15 ± 52.30%) did not reach weight gain of control mice (dark gray bar). Six independently repeated experiments. **(E–I)** Saline-perfused whole brain showed areas of hemorrhage in Notch4*^tetEC^ and Notch4*^tetEC^;*Rbpj*^*iΔEC-het*^ brains but not in Notch4*^tetEC^;*Rbpj*^*iΔEC*^, *Rbpj*^*iΔEC*^, or negative control brains. A, anterior; P, posterior. Mice from six litters or six independently repeated experiments. **(J–N)** Hemorrhage (arrowheads) and disrupted cerebellar layers were observed by H&E histological staining in Notch4*^tetEC^ and Notch4*^tetEC^;*Rbpj*^*iΔEC-het*^ cerebellum, but not in Notch4*^tetEC^;*Rbpj*^*iΔEC*^, *Rbpj*^*iΔEC*^, or negative control cerebellum. Asterisk indicates Purkinje layer. gl, granule layer; ml, molecular layer. Two independently repeated experiments. **(O–S′)** Hypoxyprobe immunostaining showed regions of hypoxic cells (arrowheads) in Notch4*^tetEC^ (7.83 ± 2.34%, *N* = 3) and Notch4*^tetEC^;*Rbpj*^*iΔEC-het*^ (4.73 ± 3.57%, *N* = 3) cerebellum but not in Notch4*^tetEC^;*Rbpj*^*iΔEC*^ (0.79 ± 0.31%, *N* = 3), *Rbpj*^*iΔEC*^ (0.52 ± 0.30%, *N* = 3), or negative control (0.25 ± 0.05%, *N* = 3) cerebellum. Quantified in **(U).** Lectin-positive staining indicated perfused vessels. Notch4*^tetEC^ (5.06 ± 1.87%, *N* = 3); Notch4*^tetEC^;*Rbpj*^*iΔEC-het*^ (4.72 ± 1.16%, *N* = 4); Notch4*^tetEC^;*Rbpj*^*iΔEC*^ (3.96 ± 0.34%, *N* = 3); *Rbpj*^*iΔEC*^ (4.21 ± 0.37%, *N* = 3); negative control (4.00 ± 1.23%, *N* = 3). Quantified in **(V)**. Four independently repeated experiments. **(T)** Schematic indicates the cerebellar region of sagittal brain section shown in O–S′. Scale bars: 5 mm in E–I; 200 μm in J–N; 400 μm in O–S′. *P<0.05; ***P<0.001.

To test whether abrogating downstream Notch signaling in Notch4*^tetEC^ mice, even while keeping the causal Notch4*^tetEC^ “on,” induces AVM regression, we deleted *Rbpj* from ECs (*Rbpj*^*iΔEC*^) in Notch4*^tetEC^ mice. We generated *Tie2-tTA*;TRE-Notch4*;*Cdh5-CreER*^*T2*^;*Rbpj*^*flox/flox*^ (Notch4*^tetEC^;*Rbpj*^*iΔEC*^) mice and four cohorts of genetic controls: *Tie2-tTA*;TRE-Notch4* (Notch4*^tetEC^); *Tie2-tTA*;TRE-Notch4*;*Cdh5-CreER*^*T2*^;*Rbpj*^*flox/+*^ (Notch4*^tetEC^;*Rbpj*^*iΔEC-het*^); *Cdh5-CreER*^*T2*^;*Rbpj*^*flox/flox*^ (*Rbpj*^*iΔEC*^); and *Cdh5-CreER*^*T2*^;*Rbpj*^*flox/+*^ (*Rbpj*^*iΔEC-he*t^; negative controls). At birth, we withdrew tet to initiate Notch4*^tetEC^ expression; beginning at P16, we administered TAM. Kaplan–Meier analysis revealed that all Notch4*^tetEC^ mice were moribund by P36 (Fig. 2, A and B); however, very few Notch4*^tetEC^;*Rbpj*^*iΔEC*^ mice were moribund at this timepoint (Fig. 2, A and B). All Notch4*^tetEC^;^RbpjiΔEC^ mice reached moribund by around P85. Heterozygous *Rbpj* deletion also delayed time to moribundity for Notch4*^tetEC^ mice (Fig. 2, A and B). Notably, endothelial deletion of *Rbpj* alone (*Rbpj*^*iΔEC*^) resulted in moribundity in about 30% of mice by P90 (Fig. 2, A and B). These results indicate that endothelial deletion of *Rbpj* from P16 improved the health of Notch4*^tetEC^ mice.

**Figure S2.**
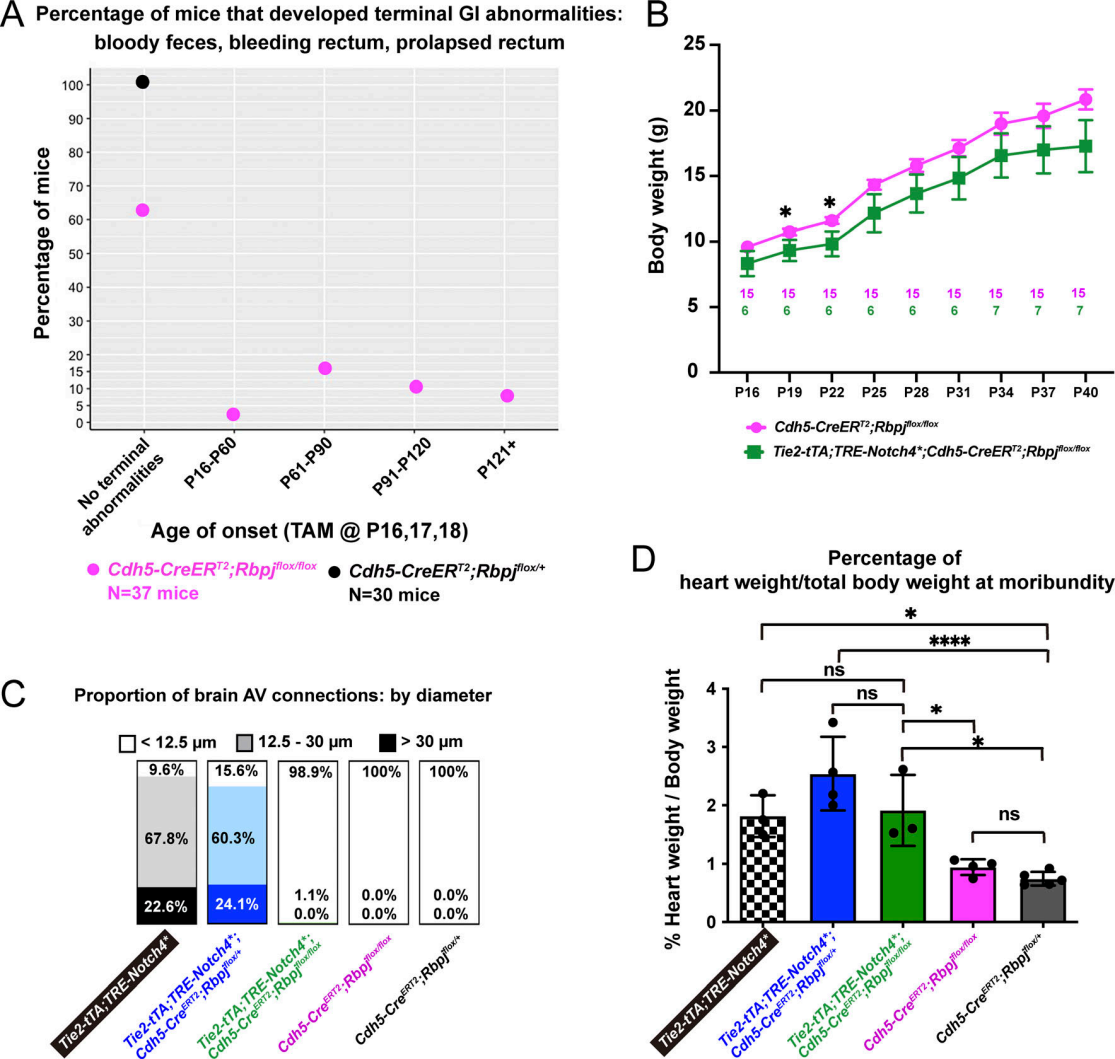
Endothelial deletion of *Rbpj* alone from P16 led to GI vascular abnormalities, while Notch4*^tetEC^ **mice with endothelial deletion of *Rbpj* from P16 decreased diameter of both moderate (12–30 μm) and severe (>30.0 μm) AV shunts, but did not normalize cardiomegaly. (A)** In total, 37.8% of *Rbpj*^*iΔEC*^ mice (14/37 mice) displayed terminal GI abnormalities that necessitated immediate euthanasia. Of those, 2.7% displayed a terminal phenotype by P60; 16.2% between P61 and P90; 10.8% between P91 and P120; 8.1% after P121. No terminal GI phenotype was detected in 62.2% of the *Rbpj*^*iΔEC*^ mice and in 100% of control mice. Nine independently repeated experiments. **(B)** By P22, Notch4*^tetEC^ mice with endothelial deletion of *Rbpj* from P16 (*Tie2-tTA*;TRE-Notch4*;*Cdh5-CreER*^*T2*^;*Rbpj*^*flox/flox*^) gained less body weight than mice with *Rbpj* deletion alone (*Cdh5-CreER*^*T2*^;*Rbpj*^*flox/flox*^). Six independently repeated experiments. Pink and green numbers in the graph refer to the number of mice weighed at each timepoint. **(C)** Normal AV connections (capillary-diameter) were defined as <12.5 μm diameter (white box); moderately enlarged AV connections were defined as 12.5–30 μm diameter (light shaded box); severely enlarged AV connection were defined as >30 μm diameter (dark shaded box). The majority of AV connection diameters were enlarged, either moderately or severely, in Notch4*^tetEC^ (90.4% total enlarged) and Notch4*^tetEC^;*Rbpj*^*iΔEC-het*^ (84.4% total enlarged) brains. In Notch4*^tetEC^;*Rbpj*^*iΔEC*^ brains, 98.9% of AV connections were normal diameter. In *Rbpj*^*iΔEC*^ and negative control mice, 100% of AV connections were normal (<12.5 μm) diameter. Proportions of AV connections with diameters <12.5 μm, 12.5–30.0 μm, or >30.0 μm were as follows: Notch4*^tetEC^ mice, 9.6% < 12.5 μm; 67.8% 12.5–30.0 μm; 22.6% > 30.0 μm; Notch4*^tetEC^;*Rbpj*^*iΔEC-het*^ mice, 15.6% < 12.5 μm; 60.3% 12.5–30.0 μm; 24.1% > 30.0 μm; Notch4*^tetEC^;*Rbpj*^*iΔEC*^ mice, 98.9% < 12.5 μm; 1.1% 12.5–30.0 μm; 0% > 30.0 μm; *Rbpj*^*iΔEC*^ mice, 100% < 12.5 μm; 0% 12.5–30.0 μm; 0% > 30.0 μm; negative controls, 100% < 12.5 μm; 0% 12.5–30.0 μm; 0% > 30.0 μm. Eight independently repeated experiments. **(D)** The percentage of heart weight (g) over total body weight (g), as evidence for cardiomegaly, was measured for each cohort and compared among cohorts: checkered column, Notch4*^tetEC^ (*N* = 3; 1.82 ± 0.35%); blue column, Notch4*^tetEC^;*Rbpj*^*iΔEC-het*^ (*N* = 4; 2.66 ± 0.71%); green column, Notch4*^tetEC^;*Rbpj*^*iΔEC*^ (*N* = 3; 1.91 ± 0.61%); pink column, *Rbpj*^*iΔEC*^ (*N* = 4; 0.94 ± 0.17%); dark gray column, negative control (*N* = 5; 0.79 ± 0.14%). Evidence for cardiomegaly, seen in Notch4*^tetEC^ mice, was not resolved by endothelial *Rbpj* deletion (compare green bar to checkered bar). Nine independently repeated experiments. *P<0.05; ****P<0.0001.

To document overall health, we monitored mice daily for signs of distress, neurological impairment, and ill health, and we harvested brain tissue at moribundity; thus, harvest time points differ among genetic cohorts. We analyzed total body weight at P16 and at moribund. At P16, no differences in body weight were seen (Fig. 2 C). At moribund, Notch4*^tetEC^ had little net change in body weight, similar to Notch4*^tetEC^;*Rbpj*^*iΔEC-het*^ mice (Fig. 2 D). By contrast, Notch4*^tetEC^;*Rbpj*^*iΔEC*^ mice gained significantly more weight than Notch4*^tetEC^; however, Notch4*tetEC;*Rbpj*^*iΔEC*^ mice did not gain as much weight as negative controls (*Rbpj*^*iΔEC-het*^; Fig. 2 D). Notably, *Rbpj*^*iΔEC*^ mice gained less body weight than negative controls (Fig. 2 D). However, when we tracked body weight changes daily from P16 TAM administration (rather than assessing change between P16 and the moribundity timepoint), we noticed that by P25, Notch4*^tetEC^;*Rbpj*^*iΔEC*^ mice were gaining weight comparably to *Rbpj*^*iΔEC*^ mice (Fig. S2 B). As a possible cause for decreased body weight gain, we found severe vascular abnormalities associated with the gastrointestinal (GI) tract in all *Rbpj*^*iΔEC*^ mice examined. In addition to enlarged, tortuous vessels in all mice analyzed, 37.8% of *Rbpj*^*iΔEC*^ mice developed terminal GI ailments—bloody feces, bleeding rectum, prolapsed rectum—that necessitated immediate euthanasia (Fig. S2 A). These data show that endothelial deletion of *Rbpj* improved the overall health of Notch4*^tetEC^ mice, while endothelial deletion of *Rbpj* alone affected animal health at a later stage.

### Endothelial deletion of *Rbpj* from P16 reduced intracerebral hemorrhage and histopathological abnormalities in Notch4*^tetEC^ mice

To assess other bAVM features, we performed gross morphological analysis of perfused brains, showing hemorrhages in moribund Notch4*^tetEC^ and Notch4*^tetEC^;*Rbpj*^*iΔEC-het*^ mice (Fig. 2, E and F), but not Notch4*^tetEC^;*Rbpj*^*iΔEC*^, *Rbpj*^*iΔEC*^ and negative control brains (Fig. 2, G–I). Histological analysis also revealed red blood cell infiltration of brain parenchyma, or hemorrhage, in Notch4*^tetEC^ and Notch4*^tetEC^;*Rbpj*^*iΔEC-het*^ mice (Fig. 2, J and K), but not in Notch4*^tetEC^;*Rbpj*^*iΔEC*^, *Rbpj*^*iΔEC*^, or negative controls (Fig. 2, L–N). We examined brain parenchyma for evidence of hypoxia, typical of AVM-adjacent tissue, in Notch4*^tetEC^ mice and found that large swaths of hypoxic cells were seen in Notch4*^tetEC^ and Notch4*^tetEC^;*Rbpj*^*iΔEC-het*^ brain tissue (Fig. 2, O, P′, and U), but not in Notch4*^tetEC^;*Rbpj*^*iΔEC*^, *Rbpj*^*iΔEC*^, or negative control tissue (Fig. 2, Q–S′, and U). As non-patent vessels could contribute to hypoxia, we measured the percentage of lectin-perfused brain tissue in all cohorts and found no significant differences (Fig. 2, O–S′, and U). Together, our results show that endothelial deletion of *Rbpj* from P16 reduced intracerebral hemorrhage and hypoxia in Notch4*^tetEC^ brains. These findings are consistent with normalization of blood flow and restoration of tissue oxygenation, following normalization of AV connections.

### Endothelial deletion of *Rbpj* from P16 normalized bAVMs in Notch4*^tetEC^ mice

**Figure 3.**
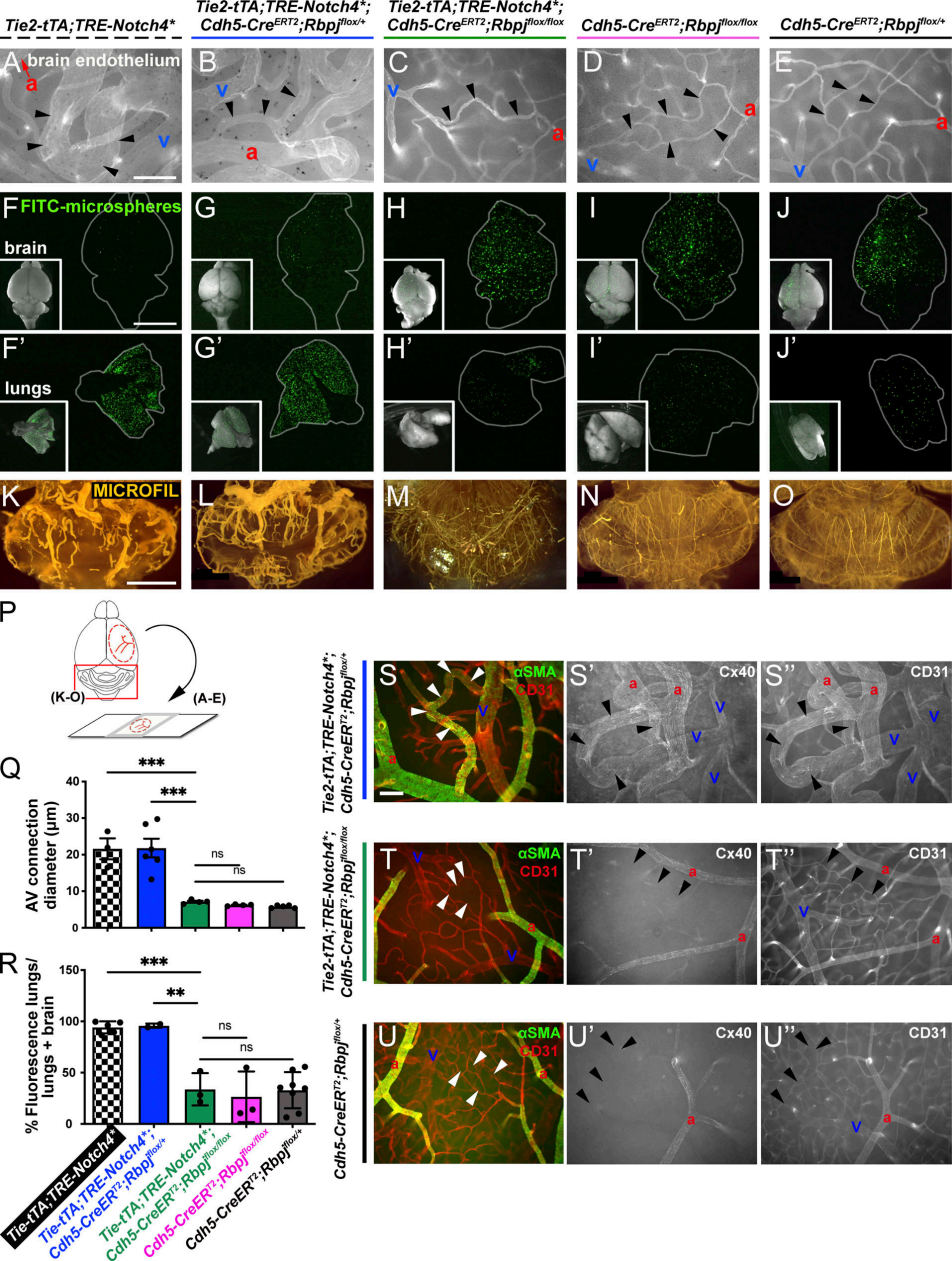
Endothelial deletion of *Rbpj* from P16 normalized brain AVM phenotypes and restored arterial identity, in AV connections and veins, in Notch4*^tetEC^ **mice. (A–E)** Whole-mount frontal cortex with FITC-lectin+ ECs to highlight vessels. We found diameters of AV connections were significantly reduced in Notch4*^tetEC^;*Rbpj*^*iΔEC*^ brains (7.14 ± 0.53 μm; *N* = 4 mice, 185 connections), as compared with Notch4*^tetEC^ (21.61 ± 4.97 μm; *N* = 3 mice, 116 connections) or Notch4*^tetEC^;*Rbpj*^*iΔEC-het*^ (21.83 ± 6.24 μm; *N* = 6 mice, 315 connections) brains. Importantly, AV connection diameters in Notch4*^tetEC^;*Rbpj*^*iΔEC*^ mice were not significantly different from either *Rbpj*^*iΔEC*^ mice (6.22 ± 0.23 μm; *N* = 4 mice, 202 connections) or negative controls (5.79 ± 0.38 μm; *N* = 5 mice, 259 connections). Arrowheads indicate AV connections. a, artery; v, vein. AV connection diameters were quantified in **(Q)**. Mice from eight litters or eight independently repeated experiments. **(F–J′)** Microspheres passed through AV shunts in Notch4*^tetEC^ (94.18 ± 5.86%, *N* = 5) and Notch4*^tetEC^;*Rbpj*^*iΔEC-het*^ (95.83 ± 2.06%, *N* = 3) brains but lodged in Notch4*^tetEC^;*Rbpj*^*iΔEC*^ (33.81 ± 28.65%, *N* = 3) brain AV connections, as well as in AV connections from *Rbpj*^*iΔEC*^ (26.54 ± 24.67%, *N* = 3) and negative control (32.91 ± 17.66%, *N* = 8) brains. Microsphere passage quantified in **(R)**. Mice from seven litters. **(K–O)** MICROFIL casting of cerebellar vasculature. Enlarged and tortuous vessels were cast in Notch4*^tetEC^ (*N* = 4) and Notch4*^tetEC^;*Rbpj*^*iΔEC-het*^ (*N* = 5) brains but were not readily observed in Notch4*^tetEC^;*Rbpj*^*iΔEC*^ (*N* = 4), *Rbpj*^*iΔEC*^ (*N* = 7), or negative control (*N* = 6) brains. 10 independently repeated experiments. **(P)** Schematic indicates whole brain regions shown in panels. Scale bars: 100 μm in A–E; 5 mm in F–J′; 2 mm in K–O. **(S–U″)** Whole-mount frontal cortex was immunostained against CD31 (to label ECs) and αSMA (S–U) or Cx40 (S′–U′) to label arterial ECs or CD31 (S″–U″) to label all ECs. White or black arrowheads indicate AV connections. a, artery; v, vein. In Notch4*^tetEC^;*Rbpj*^*iΔEC-he*t^ cortex, αSMA (S) and Cx40 (S′) expression extends beyond arteries, throughout AV shunts (arrowheads) and into veins. In both Notch4*^tetEC^;*Rbpj*^*iΔEC*^ and negative control cortex, arterial markers αSMA (T–U) and Cx40 (T′–U′) are expressed in arteries but not in AV connections (arrowheads) or veins. *N* = 3 mice for each genotype. Three independently repeated experiments. Scale bars: 100 μm. **P<0.01; ***P<0.001.

We assessed if *Rbpj* deletion in Notch4*^tetEC^ mice could attenuate features of bAVM and found that AV connection diameters were reduced in Notch4*^tetEC^;*Rbpj*^*iΔEC*^ brains as compared with Notch4*^tetEC^ or Notch4*^tetEC^;*Rbpj*^*iΔEC-het*^ brains (Fig. 3, A–C, and Q), but AV connection diameters in Notch4*^tetEC^;*Rbpj*^*iΔEC*^ mice were not significantly different from either *Rbpj*^*iΔEC*^ mice or negative controls (Fig. 3, C–E, and Q). We quantified the proportion of AV connections with diameters <12.5 μm (normal), 12.5–30.0 μm (moderate), or >30.0 μm (severe). The majority of AV connection diameters were enlarged, either moderately or severely, in Notch4*^tetEC^ (90.4%) and Notch4*^tetEC^;*Rbpj*^*iΔEC-het*^ (84.4%) brains. In Notch4*^tetEC^;*Rbpj*^*iΔEC*^, *Rbpj*^*iΔEC*^, and negative controls, 98.9, 100, and 100% of AV connections, respectively, were of normal diameter (Fig. S2 C). These data demonstrate that homozygous deletion of *Rbpj* from P16 ECs led to reduced AV connection diameter in Notch4*^tetEC^ mice.

We performed the microsphere passage assay to test whether endothelial deletion of *Rbpj* functionally attenuated AV shunting in Notch4*^tetEC^ mice. Microspheres passed through AV shunts in Notch4*^tetEC^ and Notch4*^tetEC^;*Rbpj*^*iΔEC-he*t^ brains (Fig. 3, F, G′, and R); however, microspheres lodged in narrow AV connections in Notch4*^tetEC^;*Rbpj*^*iΔEC*^ brains (Fig. 3, H, H′, and R), similar to *Rbpj*^*iΔEC*^ and negative controls (Fig. 3, I, J′, and R). Consistent with reduced AV shunting, vascular casting showed less vessel tortuosity and no evidence for nidus formation in Notch4*^tetEC^;*Rbpj*^*iΔEC*^ brains (Fig. 3 M) as compared with Notch4*^tetEC^ and Notch4*^tetEC^;*Rbpj*^*iΔEC-het*^ brains (Fig. 3, K and L). These results show that endothelial *Rbpj* deletion led to the normalization of not only the AV connection diameter but also, importantly, normalization of functional brain circulation in Notch4*^tetEC^ mice.

As AV shunting can lead to compensatory cardiomegaly, we documented increased heart/body weight ratio in Notch4*^tetEC^ mice when compared with negative controls (Fig. S2 D). Increased heart/body weight ratio was also measured in Notch4*^tetEC^;*Rbpj*^*iΔEC-het*^ and Notch4*^tetEC^;*Rbpj*^*iΔEC*^ mice when compared with negative controls. However, heart/body weight in *Rbpj*^*iΔEC*^ mice did not differ when compared with negative controls. These results show that heterozygous or homozygous EC-*Rbpj* deletion did not rescue cardiomegaly in Notch4*^tetEC^ mice and that endothelial deletion of *Rbpj* alone did not lead to cardiomegaly at moribundity.

### Endothelial deletion of *Rbpj* from P16 normalized arterial marker expression in Notch4*^tetEC^ mice

Notch signaling is required for arterial EC identity, and *Notch4*^tet^*^EC^ expression induces abnormal arterial marker expression in AV shunts and veins (Murphy et al., 2012; Murphy et al., 2008). To test whether endothelial deletion of *Rbpj* restores normal arterial identity in Notch4*^tetEC^ mice, we examined expression of αSMA and Connexin40 (Cx40). In Notch4*^tetEC^;*Rbpj*^*iΔEC-het*^ mice, both αSMA and Cx40 were expressed in arteries and abnormally expressed in AV shunts and veins (Fig. 3, S–S″). Endothelial deletion of *Rbpj* from P16 abolished the abnormal αSMA and Cx40 expression in Notch4*^tetEC^ AV connections and veins, while αSMA and Cx40 expression in arteries was maintained (Fig. 3, T–T″), resembling normal αSMA and Cx40 expression in negative controls (Fig. 3, U–U″). These data demonstrate that endothelial deletion of *Rbpj* from P16 normalized arterial marker expression in Notch4*^tetEC^ mice.

### Endothelial deletion of *Rbpj* from P21 alleviated severe bAVMs in Notch4*^tetEC^ mice

**Figure 4.**
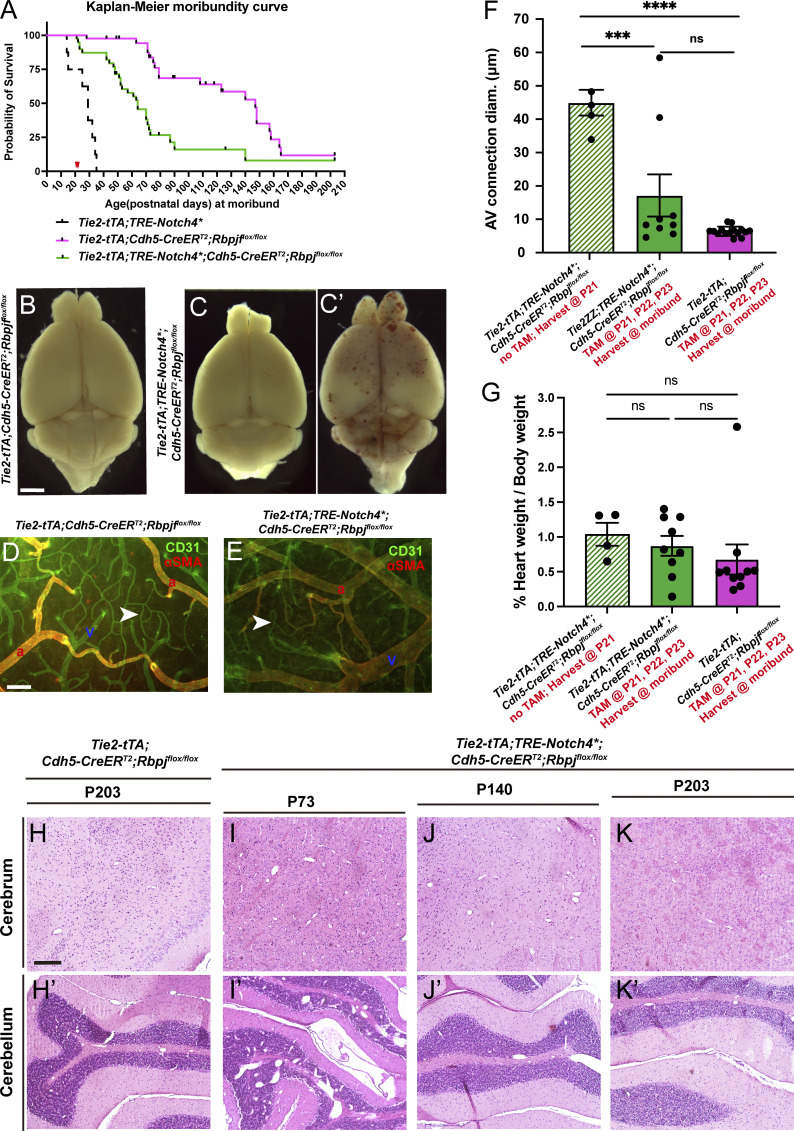
Endothelial deletion of *Rbpj* from P21 extended survival time, alleviated brain AVMs, and restored arterial marker expression in Notch4*^tetEC^ **mice. (A)** Kaplan–Meier curve. *Tie2-tTA*;TRE-Notch4*;*Cdh5-CreER*^*T2*^;*Rbpj*^*flox/flox*^ mice (green line) reached moribund later than *Tie2-tTA*;TRE-Notch4* mice (dashed black line). 12.8% (5/39) of *Tie2-tTA*;TRE-Notch4*;*Cdh5-CreER*^*T2*^;*Rbpj*^*flox/flox*^ mice moribundity by P36, 74.4% (29/39) moribundity by P90, and 12.8% (5/39) past P120. Endothelial *Rbpj* deletion alone led to 22% (9/41) moribundity by P90. Some *Tie2-tTA*;TRE-Notch4*;*Cdh5-CreER*^*T2*^;*Rbpj*^*flox/flox*^ mice lived as long as some *Tie2-tTA*;*Cdh5-CreER*^*T2*^;*Rbpj*^*flox/flox*^ mice (green and pink lines). Red arrowheads indicate TAM administration at P21–23. 13 independently repeated experiments. **(B–C′)** Cerebral hemorrhages were not observed in moribund mice with *Rbpj* deletion alone (*Tie2-tTA*;*Cdh5-CreER*^*T2*^;*Rbpj*^*flox/flox*^; *N* = 15), and 4/9 (44.5%) *Tie2-tTA*;TRE-Notch4*;*Cdh5-CreER*^*T2*^;*Rbpj*^*flox/flox*^ mice but observed in 55.5% (5/9) of *Tie2-tTA*;TRE-Notch4*;*Cdh5-CreER*^*T2*^;*Rbpj*^*flox/flox*^ (*N* = 9). C shows brain without hemorrhage; C′ shows brain with hemorrhage. **(D and E)** Flat-mount cortical immunostaining against CD31 and αSMA indicated capillary-like AV connections (arrowheads) in mice with Rbpj deletion alone (*Tie2-tTA*;*Cdh5-CreER*^*T2*^;*Rbpj*^*flox/flox*^; *N* = 15) and in Notch4*^tetEC^ mice, following endothelial Rbpj deletion from P21–23 (*Tie2-tTA*;TRE-Notch4*;*Cdh5-CreER*^*T2*^;*Rbpj*^*flox/flox*^; *N* = 9). a, artery; v, vein. Scale bars: 2 mm in B–C′, 100 μm in D and E. **(F)** Quantification of AV connections diameter. Mice of indicated genotypes were injected with or without TAM, and brain tissues were harvested at P21 or at moribundity (red annotation on x-axis legends). Endothelial deletion of *Rbpj* from P21 led to decreased brain AV diameter in *Tie2-tTA*;TRE-Notch4*;*Cdh5-CreER*^*T2*^; *Rbpj*^*flox/flox*^ (*N* = 9; green column), as compared to the initial (prior to TAM) P21 timepoint in Notch4*^tetEC^ mice (*Tie2-tTA*;TRE-Notch4*;*Cdh5-CreER*^*T2*^;*Rbpj*^*flox/flox*^) mice at P21 (*N* = 5; striped green column). AV connection diameter in *Tie2-tTA*;TRE-Notch4*;*Cdh5-CreER*^*T2*^; *Rbpj*^*flox/flox*^ post-TAM (*N* = 9; green column) were not significantly different from endothelial deletion of *Rbpj* alone (*Tie2-tTA*;*Cdh5-CreER*^*T2*^; *N* = 15; pink column). One-way ANOVA. ****P < 0.0001, ***P = 0.0006, ns P = 0.074. **(G)** Percent of heart weight/body weight was not significantly changed at moribundity between Notch4*^tetEC^ mice (striped green column), Notch4*^tetEC^ mice following endothelial *Rbpj* deletion from P21 (*Tie2-tTA*;TRE-Notch4*;*Cdh5-CreER*^*T2*^;*Rbpj*^*flox/flox*^; *N* = 9; green column), and mice with *Rbpj* deletion alone (*Tie2-tTA*;*Cdh5-CreER*^*T2*^;*Rbpj*^*flox/flox*^; *N* = 10; pink column). P = 0.472. **(H–K′)** Histological analysis of cerebrum and cerebellum over time. *Tie2-tTA*;*Cdh5-CreER*^*T2*^;*Rbpj*^*flox/flox*^ mice from eight independently repeated experiments and *Tie2-tTA*;TRE-Notch4*;*Cdh5-CreER*^*T2*^;*Rbpj*^*flox/flox*^, mice from seven independently repeated experiments. H&E-stained brain tissue showed that *Tie2-tTA*;TRE-Notch4*;*Cdh5-CreER*^*T2*^;*Rbpj*^*flox/flox*^ tissue harvested at P73 had dilated vessels in the cerebrum and in intragranular and molecular layers of cerebellum (I and I′). Tissue harvested later, from *Tie2-tTA*;TRE-Notch4*;*Cdh5-CreER*^*T2*^;*Rbpj*^*flox/flox*^ mice surviving to P140 (J and J′) or P203 (K and K′) were similar to controls (H and H′). Scale bars: 200 μm in H. ***P<0.001; ****P<0.0001.

To determine whether endothelial deletion of *Rbpj* can alleviate severe Notch4*^tetEC^ AVM, we administered TAM from P21 in Notch4*^tetEC^;*Rbpj*^*iΔEC*^ and *Rbpj*^*iΔEC*^ (control) mice. Kaplan–Meier moribundity curve showed 100% of Notch4*^tetEC^ mice were moribund by P36 (Fig. 4 A). Endothelial *Rbpj* deletion from P21 led to only 12.8% moribundity of Notch4*^tetEC^ mice by P36 and 74.4% by P90 (Fig. 4 A). Endothelial *Rbpj* deletion alone led to 22% moribundity by P90 (Fig. 4 A). By comparison, all Notch4*^tetEC^;*Rbpj*^*iΔEC*^ mice with TAM from P16 reached moribund by around P85 (Fig. 2). Because fewer Notch4*^tetEC^;*Rbpj*^*iΔEC*^ mice reached moribundity by P85 following P21 TAM administration rather than P16 TAM administration, this suggests that later *Rbpj* deletion was more beneficial for animal health. These data show that endothelial deletion of *Rbpj* from P21 extended time to moribundity in Notch4*^tetEC^ mice.

We next analyzed the effect of endothelial deletion of *Rbpj* from P21 on intracerebral hemorrhages, AV shunts, αSMA expression, and histopathologies. Five out of nine Notch4*^tetEC^;*Rbpj*^*iΔEC*^ displayed hemorrhage but not the remaining (4/9) Notch4*^tetEC^;*Rbpj*^*iΔEC*^ or any *Rbpj*^*iΔEC*^ mice (Fig. 4, B–C′). αSMA was expressed in arterial vessels but absent from venous vessels in Notch4*^tetEC^;*Rbpj*^*iΔEC*^, which was comparable with αSMA expression in *Rbpj*^*iΔEC*^ mice (Fig. 4, D and E). Mean diameter of CD31^+^ AV connections was decreased in Notch4*^tetEC^;*Rbpj*^*iΔEC*^ mice as compared to Notch4*^tetEC^ mice, and it did not differ significantly from *Rbpj*^*iΔEC*^ (Fig. 4 F). Recovery of brain tissue after P21 *Rbpj* deletion was further revealed by histological analysis, which showed that over time, brain tissue from Notch4*^tetEC^;*Rbpj*^*iΔEC*^ mice had fewer dilated vessels as compared with Notch4*^tetEC^ mice or Notch4*^tetEC^;*Rbpj*^*iΔEC*^ mice that reached moribundity earlier (Fig. 4, H–K′). As Notch4*^tetEC^ mice develop enlarged hearts, we measured the percent of heart weight/body weight in Notch4*^tetEC^;*Rbpj*^*iΔEC*^ and *Rbpj*^*iΔEC*^ mice. We found no changes between Notch4*^tetEC^ mice, Notch4*^tetEC^;*Rbpj*^*iΔEC*^ mice, and *Rbpj*^*iΔEC*^ mice (Fig. 4 G). These results indicate that endothelial deletion of *Rbpj* from P21 normalized Notch4*^tetEC^-induced severe AVM and suggest that downstream blockade of the activating pathway can lead to AVM regression.

**Figure S3.**
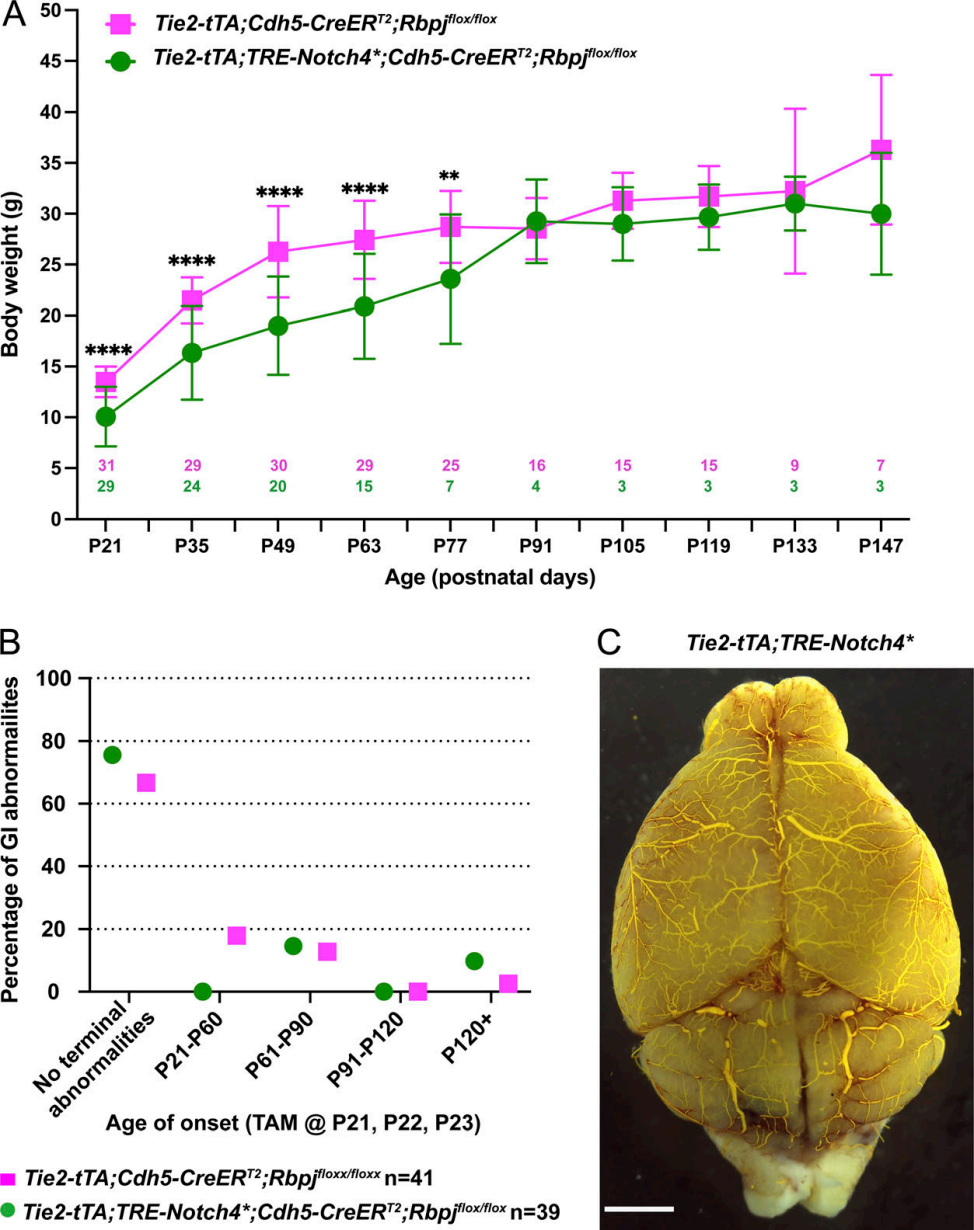
Notch4*^tetEC^ **mice with endothelial deletion of *Rbpj* from P21 gained less body weight, but both cohorts experienced similar incidence of GI abnormalities. (A)** By P77, Notch4*^tetEC^ mice with endothelial deletion of *Rbpj* from P21 (*Tie2-tTA*;TRE-Notch4*;*Cdh5-CreER*^*T2*^;*Rbpj*^*flox/flox*^) gained less body weight than mice with *Rbpj* deletion alone (*Tie2-tTA*;*Cdh5-CreER*^*T2*^;*Rbpj*^*flox/flox*^). ****P < 0.0001, **P = 0.001. **(B)** GI abnormalities were present at similar incidence in both *Tie2-tTA*;TRE-Notch4*;*Cdh5-CreER*^*T2*^;*Rbpj*^*flox/flox*^ and *Tie2-tTA*;*Cdh5-CreER*^*T2*^;*Rbpj*^*flox/flox*^ (control) mice. 13 independently repeated experiments. **(C)** One *Tie2-tTA*;TRE-Notch4* mouse out of eight after P120 tet off/Notch4* ON was moribund at P138 with hemorrhage, particularly in cerebellum, but no obvious AVM. Scale bar: 2 mm.

To gauge overall health, we tracked body weight and found that while Notch4*^tetEC^;*Rbpj*^*iΔEC*^ mice weighed less than *Rbpj*^*iΔEC*^ counterparts (Fig. S3 A) at P21, by 3 wk after *Rbpj* deletion (P42), body weight between the two cohorts was similar, and this similarity continued until P126 when Notch4*^tetEC^;*Rbpj*^*iΔEC*^ mice reached moribundity. However, overall health was not completely restored in Notch4*^tetEC^;*Rbpj*^*iΔEC*^ mice. We monitored GI health in both cohorts and found 1–15% of mice with rectal bleeding and/or prolapsed rectum between P21 and P120+ (Fig. S3 B). Our findings represent a novel proof of concept for targeting a downstream mediator of the causal Notch signaling pathway, even during advanced stages of pathogenesis, and for triggering the regression of AVM in mice. These data also show that functionally, Notch4 activates Notch canonical signaling through Rbpj in vascular endothelium. However, while this study provides a novel strategy by which targeting Notch signaling can normalize AVM pathologies, targeting *Rbpj* itself is not ideal as it affected animal health at a later stage. This study may inspire future development of strategies to inhibit Notch signaling without compromising animal health.

### Virtually no relapse after complete AVM regression, despite re-expression of Notch4*^tetEC^ in adult mice

**Figure 5.**
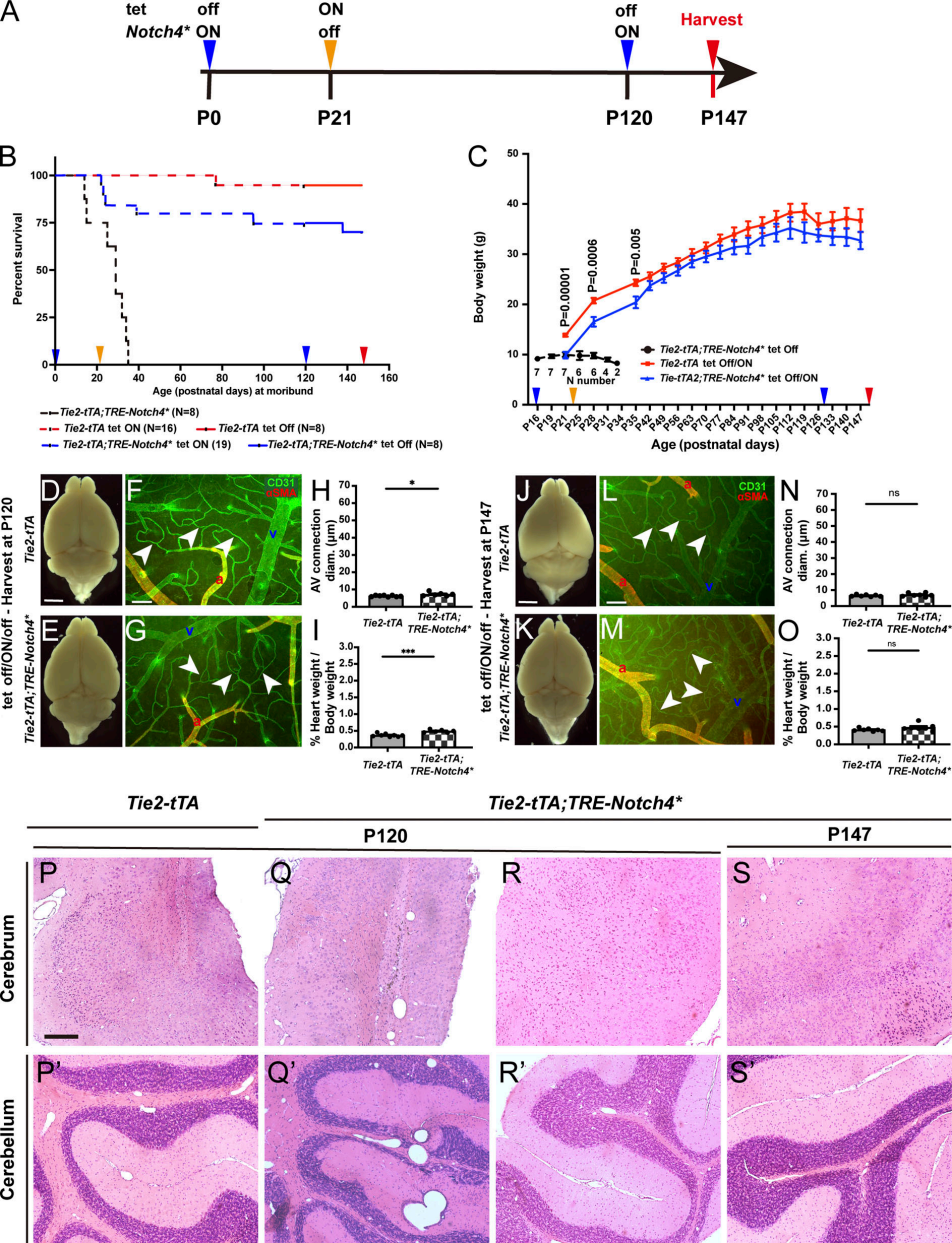
Virtually no relapse after re-expression of Notch4*^tetEC^ **in P120 mice, following complete normalization of AVM. (A)** Experimental timeline for expression (tet off/Notch4* ON) and suppression (tet ON/Notch4* off) of Notch4*^tetEC^ via administration of one dose (i.p. injection) of tet and Dox food (hereafter tet) and for tissue harvest. Arrowheads indicate experimental timepoints in B and C. **(B)** Kaplan–Meier moribundity curve shows that time to moribundity was extended in *Tie2-tTA*;TRE-Notch4* mice by tet off/ON/off treatment with Dox food (dashed or solid blue line), as compared to mice without Dox food (tet off; dashed black line). Specifically, 15.8% (3/19) of *Tie2-tTA*;TRE-Notch4* mice did not survive beyond P24, likely due to illness, 5.3% (1/19) died at P39, 5.3% (1/19) died at P95, and 73.7% (14/19) survived through P120 (dashed blue line). 6.3% (1/16) of *Tie2-tTA* mice died at P77 and 93.7% (15/16) survived through P120 (dashed red line). After P120 tet off/Notch4* ON, 12.5% (1/8) of *Tie2-tTA*;TRE-Notch4* mice died at P138, and 87.5% (7/8) survived through P147 (solid blue line). 14.3% (1/7) of negative controls died at P77 and 85.7% (6/7) survived through P147 (solid red line). Mice came from eight litters. **(C)** Body weight changes of mice in B, and P values indicate the comparison between *Tie2-tTA* tet off/ON and *Tie2-tTA*;TRE-Notch4*. Notch4*^tetEC^ mice with tet off (black line) failed to gain weight and did not survive past P34. Notch4*^tetEC^ mice with tet off/ON/off (blue line) gained weight and kept pace with negative controls (red line). **(D–O)** Analysis of brain pathologies in Notch4*^tetEC^ mice and negative controls, all of which were subject to the tet off/ON/off (Notch4* ON/off/ON) experimental regimen. Tissue harvest was at P120 (D–I) or P147 (J–O). Four independently repeated experiments. **(D and E)** No hemorrhages were detected in either cohort by P120. **(F–H)** Frontal cortices were immunostained against CD31 and αSMA. AV connection (arrowheads) diameter was slightly increased in P120 Notch4*^tetEC^ mice (7.3 ± 1.1, *N* = 6), as compared to negative controls (6.2 ± 0.41, *N* = 8). P = 0.029. However, the values were similar to capillary-diameter values (∼5–7 μm). **(I)** Higher heart weight/body weight ratio was found in P120 *Tie2-tTA*;TRE-Notch4* mice (0.49 ± 0.043, *N* = 6) at P120, as compared to negative control (*Tie2-tTA*) mice (0.37 ± 0.045, *N* = 8). P = 0.0002. **(J and K)** No hemorrhages were detected in either cohort by P147. **(L–N)** Frontal cortices were immunostained against CD31 and αSMA. AV connection diameter (arrowheads) was not significantly different in P147 Notch4*^tetEC^ mice (*N* = 7), as compared to negative controls (*N* = 7). P = 0.3533. **(O)** No change in heart weight/body weight ratio was found in P147 *Tie2-tTA*;TRE-Notch4* mice (*N* = 7) at P120, as compared to negative control (*Tie2-tTA*) mice (*N* = 7). P = 0.1164. a, artery; v, vein. Scale bars: 2 mm in D and E, J and K, 100 μm in F, G, L, and M. **(P–S′)** Histological analysis of cerebrum and cerebellum, over time. On P120, two out of four *Tie2-tTA*;TRE-Notch4* mice exhibited focal deposits of hemosiderin and macrophages and in the posterior superior cerebral cortex region, evidence of prior damage/hemorrhage undergoing repair (P and Q). The same two *Tie2-tTA*;TRE-Notch4* mice also exhibited dilated vessels in the cerebellum compared to the control mice (P′ and Q′). No obvious lesions were found in the other two *Tie2-tTA*;TRE-Notch4*mice (represented in R and R′). No new hemorrhages were observed in any of the mice examined. **(S and S′)** On P147, all five *Tie2-tTA*;TRE-Notch4* mice were indistinguishable in their histopathological features, compared to *Tie2-tTA* control mice. P120 and P147 mice from four independently repeated experiments. Scale bar: 200 μm in P–S′. *P<0.05; ***P<0.001.

To test whether re-expression of the causal Notch4*^tetEC^ triggered relapse of AVM formation, we designed the following experimental paradigm (Fig. 5 A). (1) We bred *Tie2-tTA*;TRE-Notch4* (Notch4*^tetEC^) and *Tie2-tTA* (controls) and withdrew tet at birth to express Notch4*^tetEC^; (2) we allowed advanced AVM to develop until P21, at which time Notch4*^tetEC^ was suppressed by tet injection and doxycycline (Dox) chow; (3) we allowed 100 d on Dox chow for complete AVM regression; (4) at P120, we removed Dox chow to re-express Notch4*^tetEC^; and (5) we harvested brain tissue 4 wk later at P147 to evaluate the AVM phenotype. Kaplan–Meier analysis revealed that 100% of Notch4*^tetEC^ mice were moribund by P36 (Fig. 5 B). By contrast, only 23% Notch4*^tetEC^ mice (tet off/ON/off paradigm) died by P36 (of those, all died shortly after P21 and by P24), indicating that these mice were too sick to be rescued, and 73.68% survived to P120. Once mice survived beyond the initial treatment time, 87.5% (14/16) mice live beyond 120 days (Fig. 5 B). Suppression of Notch4*^tetEC^ from P21 (tet off/ON/off paradigm) permitted body weight gain similar to *Tie2-tTA* mice (Fig. 5 C). We assessed the degree of bAVM regression by P120, following Notch4*^tetEC^ induction at birth and suppression at P21. Whole brains showed no evidence for hemorrhage in Notch4*^tetEC^ mice (tet off/ON/off paradigm) at P120 as compared with controls (Fig. 5, D and E), and αSMA expression was limited to arterial vessel segments in Notch4*^tetEC^, as in controls (tet off/ON/off paradigm; Fig. 5, F and G). CD31^+^ AV connections (Fig. 5, F–H) and percent heart weight/body weight (Fig. 5 I) in Notch4*^tetEC^ were slightly increased when compared with *Tie2-tTA* controls (tet off/ON/off paradigm). This confirms that suppression of Notch4*^tetEC^ from P21 nearly normalizes severe AVMs by P120.

Finally, we asked whether AVMs relapse, following reinduction of Notch4*^tetEC^ expression at P120. To our surprise, 87.5% (7/8) mice exhibited further improvement at P147 with no detectable hemorrhage (Fig. 5, J and K), vascular abnormalities (Fig. 5, L–N), histopathologies (Fig. 5, P–S′), or enlarged heart weight/body weight ratio (Fig. 5 O). At P138, one mouse was found non-responsive, and casting did not show obvious AVMs in the cortex, though evidence of abnormal vessels and hemorrhage was found in the cerebellum (Fig. S3 C). It is possible that brain tissues were not completely normalized at P120 in this case. Thus, a longer recovery was needed to achieve continued health in this mouse. These results suggest that AVM was not reinitiated after P120, following recovery from P21. Our data show that removal of the causal transgene resolved pathologies associated with Notch4*^tetEC^ AVM and that after near-complete resolution of pathologies, even if the causal transgene was switched back on, there is no AVM relapse. These data are also consistent with our previous finding that expression of Notch4*^tetEC^ in immature, but not mature, ECs induces hallmarks of bAVM (Carlson et al., 2005) and support the idea that mature brain vasculature is not susceptible to Notch4*^tetEC^-induced AVM formation.

Collectively, our findings make an important advance, demonstrating both AVM regression and tissue reperfusion in Notch4*^tetEC^ mice by targeting a downstream signaling component or by causal transgene reversal. These data show that endothelial Rbpj is critical for the initiation and maintenance of Notch4*^tetEC^-induced brain AVM in mice and that endothelial Rbpj may be targeted during different stages of AVM pathogenesis. After complete recovery, reintroducing the causal gene does not lead to relapse later in life.

## Materials and methods

### Mice

The following mouse transgenes were used: *Tie2-tTA* (Carlson et al., 2005), TRE-Notch4* (Carlson et al., 2005), *Cdh5*(*PAC*)*-CreER*^*T2*^ (or *Cdh5-CreER*^*T2*^ herein; Sorensen et al., 2009; Wang et al., 2010; Ralf Adams, Max Planck Institute for Molecular Biomedicine, Münster, Germany), and *Rbpj*^*flox*^ (*Rbpsuh*^*flox*^; Tanigaki et al., 2002; Tasuku Honjo, Kyoto University, Kyoto, Japan). We combined two independent, inducible genetic systems: tet-OFF (withdrawal of tet led to Notch4*^tetEC^ activation) and CreER-lox*P* (TAM administration led to *Rbpj*^*iΔEC*^ deletion). We generated *Tie2-tTA*;TRE-Notch4*;*Cdh5-CreER*^*T2*^;*Rbpj*^flox/flox^ (referred to as Notch4*^tetEC^;*Rbpj*^*iΔEC*^ mice) and four cohorts of genetic controls: *Tie2-tTA*;TRE-Notch4* (Notch4*^tetEC^); *Tie2-tTA*;TRE-Notch4*;*Cdh5-CreER*^*T2*^;*Rbpj*^*flox/+*^ (Notch4*^tetEC^;*Rbpj*^*iΔEC-het*^); *Cdh5-CreER*^*T2*^;*Rbpj*^*flox/flox*^ (*Rbpj*^*iΔEC*^); and *Cdh5-CreER*^*T2*^;*Rbpj*^*flox/+*^ (negative controls in Figs. 2, 3, and S2). In Figs. 1, 5, and S1, C and D, *Tie2-tTA* mice were used as negative controls. In Figs. 4, S1, E–M, and S3, *Tie2-tTA*;*Cdh5-CreER*^*T2*^;*Rbpj*^*flox/flox*^ mice were used as negative controls. Mice used in the tet-inducible system alone (Figs. 1, 5, and S1, C and D) were maintained on FVBN genetic background. Mice used in the tet- and TAM-inducible combined systems (Figs. 2, 3, 4, S1, E–M, S2, and S3) were maintained on a mixed genetic background. At birth, we withdrew tet to initiate Notch4*^tetEC^ expression; beginning at P16, we administered TAM for 3 consecutive days. For tet-regulated transgene expression (tet-repressible), Notch4*^tetEC^ was repressed by administering Dox (200 mg/kg; Bio-Serv) in chow ad libitum; prior to plugging, mating pairs were switched to regular chow and were administered tet in sweetened drinking water (0.5 mg/ml tet + 50 mg/ml sucrose; Sigma-Aldrich); pregnant dams received tet water throughout gestational period. At birth, dams and pups were switched to regular drinking water to induce Notch4*^tetEC^ transgene expression. For CreER^T2^-mediated recombination, 0.5 mg TAM (Sigma-Aldrich)/50 μl peanut oil (Planters) was injected i.p. once daily at P16–P18 and P21–P23. For Notch4*^tetEC^ suppression, mice were injected i.p. with 0.5 mg tet and administered Dox (200 mg/kg; Bio-Serv) in chow ad libitum. Animals were maintained and treated in accordance with the University of California, San Francisco Institutional Animal Care and Use Committee guidelines.

### Vascular staining and immunostaining

To stain vascular endothelium, we perfused 100 μg FITC-conjugated *Lycopersicon esculentum* (Tomato) Lectin (Vector Labs)/150 μl PBS by intravenous injection into the inferior vena cava of anesthetized mice. Immunostaining was performed, according to our published method (Murphy et al., 2012), with the following modifications: anti-CD31 (1:500; BD Pharmingen), anti-αSMA (1:1,000; Sigma-Aldrich), and anti-Connexin40 (1:400; Alpha Diagnostic International).

### Vascular casting

Casts of brain vasculature were prepared as follows: anesthetized mice were exsanguinated by transcardial perfusion of PBS; MICROFIL:diluent:curing agent (4:5:1) was transcardially perfused; MICROFIL cast cured at room temperature for 45 min; brain tissue was harvested, imaged, or dehydrated in ethanol series, and cleared in methyl salicylate, according to manufacturer’s instructions. Images were captured using dissection scope and Leica LAS software.

### Microsphere passage assay

Mice were anesthetized using isoflurane/oxygen and the left common carotid artery was surgically exposed. 75 μl of 15 μm FluoSpheres, green (450–480 nm; Invitrogen) were injected directly into the left common carotid artery and circulated for 1 min. Brain and lung tissues were harvested and imaged using a fluorescent/brightfield dissection microscope (Leica) and MicroManager software.

### Hypoxia assay

Hypoxia assay was followed according to the manufacturer’s protocol (HPi-100, HPI, Inc.). Briefly, mice were injected i.p. with 60 mg/kg body weight of pimonidazole HCl in 0.9% saline. After 90 min, mice were intravenously perfused with 100 μg FITC-lectin (Vector Labs)/150 μl PBS and then transcardially perfused with 1% PFA; brain tissue was harvested and prepared for cryosectioning. Immunostaining against 1:50 dilution of MAb1 (4.3.11.3 mouse IgG_1_ anti-pimonidazole monoclonal antibody; HPI, Inc.) was performed according to manufacturer’s recommendations using Cy3 donkey anti-mouse secondary antibody (Jackson ImmunoResearch). VECTASHIELD Mounting Media with DAPI (Vector Labs) was applied. Images were captured using an upright fluorescent microscope and SlideBook software.

### Gross morphology and histology

For whole-brain imaging to assess indication of hemorrhage, anesthetized mice were exsanguinated by transcardial perfusion of PBS, followed by 1% PFA. Brains were harvested and imaged using a dissection microscope and Leica LAS software. Standard H&E staining on paraffin-embedded tissue sections was performed in the lab and by the Gladstone Institutes Histology and Light Microscopy Core. Images were captured using an upright light microscope and ZEN software (Zeiss).

### Quantification using ImageJ

ImageJ software was used to measure (1) diameters of AV connections at their narrowest; (2) hypoxyprobe+ area per total area of tissue examined; and (3) perfused lectin+ area per total area of tissue section examined. Measurement data were acquired from 12-μm-thick tissue sections.

### Statistical analysis

Data are shown as mean ± SD. An unpaired Student’s *t* test with Welch’s correction or one-way ANOVA with Tukey’s multiple comparison test was used to analyze variance among experimental groups. P < 0.05 was considered significant. Data in the figures are annotated as follows: *P < 0.05; **P < 0.01; and ***P < 0.001. Prism software (Graph Pad) or R Statistical Software was used to generate data graphs and perform statistical analyses.

### Online supplemental material

Fig. S1 shows additional characterizations of Notch4-induced brain AVM established by P16 and P21. Fig. S2 shows the incidence of gastrointestinal vascular abnormalities in *Rbpj*^*iΔEC*^ mice, as well as the normalization of AV diameter, but not of cardiomegaly, in Notch4*^tetEC^ mice with endothelial deletion of *Rbpj* from P16. Fig. S3 shows the incidence of gastrointestinal vascular abnormalities and tracking of body weight gain in Notch4*^tetEC^ mice with endothelial deletion of *Rbpj* from P21.

## References

[bib1] Acker, T., H. Beck, and K.H. Plate. 2001. Cell type specific expression of vascular endothelial growth factor and angiopoietin-1 and -2 suggests an important role of astrocytes in cerebellar vascularization. Mech. Dev. 108:45–57. 10.1016/S0925-4773(01)00471-3 11578860

[bib2] Bayrak-Toydemir, P., J. McDonald, N. Akarsu, R.M. Toydemir, F. Calderon, T. Tuncali, W. Tang, F. Miller, and R. Mao. 2006. A fourth locus for hereditary hemorrhagic telangiectasia maps to chromosome 7. Am. J. Med. Genet. A. 140:2155–2162. 10.1002/ajmg.a.31450 16969873

[bib3] Bendok, B.R., R.J. Rahme, S.G. Aoun, T.Y. El Ahmadieh, N.E. El Tecle, H.H. Batjer, and A.J. Fishman. 2014. Enhancement of the subtemporal approach by partial posterosuperior petrosectomy with hearing preservation. Neurosurgery. 10:191–199. 10.1227/NEU.0000000000000300 24476903

[bib4] Benedito, R., C. Roca, I. Sörensen, S. Adams, A. Gossler, M. Fruttiger, and R.H. Adams. 2009. The notch ligands Dll4 and Jagged1 have opposing effects on angiogenesis. Cell. 137:1124–1135. 10.1016/j.cell.2009.03.025 19524514

[bib5] Benzinou, M., F.F. Clermont, T.G. Letteboer, J.H. Kim, S. Espejel, K.A. Harradine, J. Arbelaez, M.T. Luu, R. Roy, D. Quigley, . 2012. Mouse and human strategies identify PTPN14 as a modifier of angiogenesis and hereditary haemorrhagic telangiectasia. Nat. Commun. 3:616. 10.1038/ncomms1633 22233626 PMC3509798

[bib6] Bray, S.J. 2016. Notch signalling in context. Nat. Rev. Mol. Cell Biol. 17:722–735. 10.1038/nrm.2016.94 27507209

[bib7] Burger, B., S. Uhlhaas, E. Mangold, P. Propping, W. Friedl, D. Jenne, G. Dockter, and W. Back. 2002. Novel de novo mutation of MADH4/SMAD4 in a patient with juvenile polyposis. Am. J. Med. Genet. 110:289–291. 10.1002/ajmg.10411 12116240

[bib8] Carlson, T.R., Y. Yan, X. Wu, M.T. Lam, G.L. Tang, L.J. Beverly, L.M. Messina, A.J. Capobianco, Z. Werb, and R. Wang. 2005. Endothelial expression of constitutively active Notch4 elicits reversible arteriovenous malformations in adult mice. Proc. Natl. Acad. Sci. USA. 102:9884–9889. 10.1073/pnas.0504391102 15994223 PMC1175015

[bib52] Chapman, A.D., S. Selhorst, J. LaComb, A. LeDantec-Boswell, T. Wohl, S. Adhicary, and C.M. Nielsen. 2022. Endothelial Rbpj is required for cerebellar morphogenesis and motor control in the early postnatal mouse brain. The Cerebellum. 10.1007/s12311-022-01429-w 35716334

[bib9] Chen, W., E.J. Choi, C.M. McDougall, and H. Su. 2014. Brain arteriovenous malformation modeling, pathogenesis, and novel therapeutic targets. Transl. Stroke Res. 5:316–329. 10.1007/s12975-014-0343-0 24723256 PMC4081044

[bib10] Cole, S.G., M.E. Begbie, G.M. Wallace, and C.L. Shovlin. 2005. A new locus for hereditary haemorrhagic telangiectasia (HHT3) maps to chromosome 5. J. Med. Genet. 42:577–582. 10.1136/jmg.2004.028712 15994879 PMC1736109

[bib11] Cuervo, H., C.M. Nielsen, D.A. Simonetto, L. Ferrell, V.H. Shah, and R.A. Wang. 2016. Endothelial notch signaling is essential to prevent hepatic vascular malformations in mice. Hepatology. 64:1302–1316. 10.1002/hep.28713 27362333 PMC5261867

[bib12] Davis, R.B., K. Pahl, N.C. Datto, S.V. Smith, C. Shawber, K.M. Caron, and J. Blatt. 2018. Notch signaling pathway is a potential therapeutic target for extracranial vascular malformations. Sci. Rep. 8:17987. 10.1038/s41598-018-36628-1 30573741 PMC6302123

[bib13] Delev, D., A. Pavlova, A. Grote, A. Boström, A. Höllig, J. Schramm, R. Fimmers, J. Oldenburg, and M. Simon. 2017. NOTCH4 gene polymorphisms as potential risk factors for brain arteriovenous malformation development and hemorrhagic presentation. J. Neurosurg. 126:1552–1559. 10.3171/2016.3.JNS151731 27231971

[bib14] Fang, J.S., B.G. Coon, N. Gillis, Z. Chen, J. Qiu, T.W. Chittenden, J.M. Burt, M.A. Schwartz, and K.K. Hirschi. 2017. Shear-induced Notch-Cx37-p27 axis arrests endothelial cell cycle to enable arterial specification. Nat. Commun. 8:2149. 10.1038/s41467-017-01742-7 29247167 PMC5732288

[bib15] Florian, I.A., T.L. Timiș, G. Ungureanu, I.S. Florian, A. Bălașa, and I. Berindan-Neagoe. 2020. Deciphering the vascular labyrinth: Role of microRNAs and candidate gene SNPs in brain AVM development—literature review. Neurol. Res. 42:1043–1054. 10.1080/01616412.2020.1796380 32723034

[bib16] Friedlander, R.M. 2007. Clinical practice. Arteriovenous malformations of the brain. N. Engl. J. Med. 356:2704–2712. 10.1056/NEJMcp067192 17596605

[bib17] Fu, W., R. Huo, Z. Yan, H. Xu, H. Li, Y. Jiao, L. Wang, J. Weng, J. Wang, S. Wang, . 2020. Mesenchymal behavior of the endothelium promoted by SMAD6 downregulation is associated with brain arteriovenous malformation microhemorrhage. Stroke. 51:2197–2207. 10.1161/STROKEAHA.120.030046 32486965

[bib18] Gallione, C.J., G.M. Repetto, E. Legius, A.K. Rustgi, S.L. Schelley, S. Tejpar, G. Mitchell, E. Drouin, C.J. Westermann, and D.A. Marchuk. 2004. A combined syndrome of juvenile polyposis and hereditary haemorrhagic telangiectasia associated with mutations in MADH4 (SMAD4). Lancet. 363:852–859. 10.1016/S0140-6736(04)15732-2 15031030

[bib19] Giaimo, B.D., and T. Borggrefe. 2018. Introduction to molecular mechanisms in notch signal transduction and disease pathogenesis. Adv. Exp. Med. Biol. 1066:3–30. 10.1007/978-3-319-89512-3_1 30030819

[bib20] Hartmann, A., H. Mast, J.P. Mohr, H.C. Koennecke, A. Osipov, J. Pile-Spellman, D.H. Duong, and W.L. Young. 1998. Morbidity of intracranial hemorrhage in patients with cerebral arteriovenous malformation. Stroke. 29:931–934. 10.1161/01.STR.29.5.931 9596237

[bib21] Hellström, M., L.K. Phng, J.J. Hofmann, E. Wallgard, L. Coultas, P. Lindblom, J. Alva, A.K. Nilsson, L. Karlsson, N. Gaiano, . 2007. Dll4 signalling through Notch1 regulates formation of tip cells during angiogenesis. Nature. 445:776–780. 10.1038/nature05571 17259973

[bib22] Hill-Felberg, S., H.H. Wu, S.A. Toms, and A.R. Dehdashti. 2015. Notch receptor expression in human brain arteriovenous malformations. J. Cell. Mol. Med. 19:1986–1993. 10.1111/jcmm.12580 25846406 PMC4549049

[bib54] Hong, T., Y. Yan, J. Li, I. Radovanovic, X. Ma, Y.W. Shao, J. Yu, Y. Ma, P. Zhang, F. Ling, . 2019. High prevalence of KRAS/BRAF somatic mutations in brain and spinal cord arteriovenous malformations. Brain. 142(1):23–34. 10.1093/brain/awy307 30544177

[bib23] Johnson, D.W., J.N. Berg, M.A. Baldwin, C.J. Gallione, I. Marondel, S.J. Yoon, T.T. Stenzel, M. Speer, M.A. Pericak-Vance, A. Diamond, . 1996. Mutations in the activin receptor-like kinase 1 gene in hereditary haemorrhagic telangiectasia type 2. Nat. Genet. 13:189–195. 10.1038/ng0696-189 8640225

[bib24] Kerr, B.A., X.Z. West, Y.W. Kim, Y. Zhao, M. Tischenko, R.M. Cull, T.W. Phares, X.D. Peng, J. Bernier-Latmani, T.V. Petrova, . 2016. Stability and function of adult vasculature is sustained by Akt/Jagged1 signalling axis in endothelium. Nat. Commun. 7:10960. 10.1038/ncomms10960 26971877 PMC4793084

[bib25] Krebs, L.T., J.R. Shutter, K. Tanigaki, T. Honjo, K.L. Stark, and T. Gridley. 2004. Haploinsufficient lethality and formation of arteriovenous malformations in Notch pathway mutants. Genes Dev. 18:2469–2473. 10.1101/gad.1239204 15466160 PMC529533

[bib26] Krebs, L.T., C. Starling, A.V. Chervonsky, and T. Gridley. 2010. Notch1 activation in mice causes arteriovenous malformations phenocopied by ephrinB2 and EphB4 mutants. Genesis. 48:146–150. 10.1002/dvg.20599 20101599 PMC2849749

[bib27] Krebs, L.T., Y. Xue, C.R. Norton, J.R. Shutter, M. Maguire, J.P. Sundberg, D. Gallahan, V. Closson, J. Kitajewski, R. Callahan, . 2000. Notch signaling is essential for vascular morphogenesis in mice. Genes Dev. 14:1343–1352. 10.1101/gad.14.11.1343 10837027 PMC316662

[bib28] Letteboer, T.G., M. Benzinou, C.B. Merrick, D.A. Quigley, K. Zhau, I.J. Kim, M.D. To, D.M. Jablons, J.K.P. van Amstel, C.J. Westermann, . 2015. Genetic variation in the functional ENG allele inherited from the non-affected parent associates with presence of pulmonary arteriovenous malformation in hereditary hemorrhagic telangiectasia 1 (HHT1) and may influence expression of PTPN14. Front. Genet. 6:67. 10.3389/fgene.2015.00067 25815003 PMC4357294

[bib29] Lobov, I.B., R.A. Renard, N. Papadopoulos, N.W. Gale, G. Thurston, G.D. Yancopoulos, and S.J. Wiegand. 2007. Delta-like ligand 4 (Dll4) is induced by VEGF as a negative regulator of angiogenic sprouting. Proc. Natl. Acad. Sci. USA. 104:3219–3224. 10.1073/pnas.0611206104 17296940 PMC1805530

[bib30] McAllister, K.A., K.M. Grogg, D.W. Johnson, C.J. Gallione, M.A. Baldwin, C.E. Jackson, E.A. Helmbold, D.S. Markel, W.C. McKinnon, J. Murrell, . 1994. Endoglin, a TGF-beta binding protein of endothelial cells, is the gene for hereditary haemorrhagic telangiectasia type 1. Nat. Genet. 8:345–351. 10.1038/ng1294-345 7894484

[bib31] Murphy, P.A., T.N. Kim, G. Lu, A.W. Bollen, C.B. Schaffer, and R.A. Wang. 2012. Notch4 normalization reduces blood vessel size in arteriovenous malformations. Sci. Transl. Med. 4:117ra8. 10.1126/scitranslmed.3002670 PMC3320799 22261032

[bib32] Murphy, P.A., M.T. Lam, X. Wu, T.N. Kim, S.M. Vartanian, A.W. Bollen, T.R. Carlson, and R.A. Wang. 2008. Endothelial Notch4 signaling induces hallmarks of brain arteriovenous malformations in mice. Proc. Natl. Acad. Sci. USA. 105:10901–10906. 10.1073/pnas.0802743105 18667694 PMC2504798

[bib33] Murphy, P.A., G. Lu, S. Shiah, A.W. Bollen, and R.A. Wang. 2009. Endothelial Notch signaling is upregulated in human brain arteriovenous malformations and a mouse model of the disease. Lab. Invest. 89:971–982. 10.1038/labinvest.2009.62 19546852 PMC3095492

[bib34] Nielsen, C.M., H. Cuervo, V.W. Ding, Y. Kong, E.J. Huang, and R.A. Wang. 2014. Deletion of Rbpj from postnatal endothelium leads to abnormal arteriovenous shunting in mice. Development. 141:3782–3792. 10.1242/dev.108951 25209249 PMC4197591

[bib53] Nikolaev, S.I., S. Vetiska, E. Bonilla, E. Boudreau, S. Jauhiainen, B. Rezai Jahromi, N. Khyzha, P.V. DiStefano, S. Suutarinen, T.-R. Kiehl, . 2018. Somatic activating KRAS mutations in arteriovenous malformations of the brain. New Engl. J. Med. 378:250–261. 10.1056/NEJMoa1709449 29298116 PMC8161530

[bib35] Olivieri, C., E. Mira, G. Delù, F. Pagella, A. Zambelli, L. Malvezzi, E. Buscarini, and C. Danesino. 2002. Identification of 13 new mutations in the ACVRL1 gene in a group of 52 unselected Italian patients affected by hereditary haemorrhagic telangiectasia. J. Med. Genet. 39:E39. 10.1136/jmg.39.7.e39 12114496 PMC1735165

[bib36] Pitulescu, M.E., I. Schmidt, B.D. Giaimo, T. Antoine, F. Berkenfeld, F. Ferrante, H. Park, M. Ehling, D. Biljes, S.F. Rocha, . 2017. Dll4 and Notch signalling couples sprouting angiogenesis and artery formation. Nat. Cell Biol. 19:915–927. 10.1038/ncb3555 28714968

[bib37] Ponce, F.A., and R.F. Spetzler. 2011. Arteriovenous malformations: Classification to cure. Clin. Neurosurg. 58:10–12. 10.1227/NEU.0b013e318226a248 21916123

[bib38] Rangel-Castilla, L., J.J. Russin, E. Martinez-Del-Campo, H. Soriano-Baron, R.F. Spetzler, and P. Nakaji. 2014. Molecular and cellular biology of cerebral arteriovenous malformations: A review of current concepts and future trends in treatment. Neurosurg. Focus. 37:E1. 10.3171/2014.7.FOCUS14214 25175428

[bib39] Rutledge, W.C., N.U. Ko, M.T. Lawton, and H. Kim. 2014. Hemorrhage rates and risk factors in the natural history course of brain arteriovenous malformations. Transl. Stroke Res. 5:538–542. 10.1007/s12975-014-0351-0 24930128 PMC4139097

[bib40] Sasahara, A., H. Kasuya, H. Akagawa, H. Ujiie, O. Kubo, T. Sasaki, H. Onda, Y. Sakamoto, B. Krischek, T. Hori, and I. Inoue. 2007. Increased expression of ephrin A1 in brain arteriovenous malformation: DNA microarray analysis. Neurosurg. Rev. 30:299–305. 10.1007/s10143-007-0087-3 17576607

[bib41] Scimone, C., F. Granata, M. Longo, E. Mormina, C. Turiaco, A.A. Caragliano, L. Donato, A. Sidoti, and R. D’Angelo. 2020. Germline mutation enrichment in pathways controlling endothelial cell homeostasis in patients with brain arteriovenous malformation: Implication for molecular diagnosis. Int. J. Mol. Sci. 21:4321. 10.3390/ijms21124321 32560555 PMC7352422

[bib42] Shoemaker, L.D., A.K. McCormick, B.M. Allen, and S.D. Chang. 2020. Evidence for endothelial-to-mesenchymal transition in human brain arteriovenous malformations. Clin. Transl. Med. 10:e99. 10.1002/ctm2.99 32564509 PMC7403663

[bib43] Sörensen, I., R.H. Adams, and A. Gossler. 2009. DLL1-mediated Notch activation regulates endothelial identity in mouse fetal arteries. Blood. 113:5680–5688. 10.1182/blood-2008-08-174508 19144989

[bib44] Suchting, S., C. Freitas, F. le Noble, R. Benedito, C. Bréant, A. Duarte, and A. Eichmann. 2007. The Notch ligand Delta-like 4 negatively regulates endothelial tip cell formation and vessel branching. Proc. Natl. Acad. Sci. USA. 104:3225–3230. 10.1073/pnas.0611177104 17296941 PMC1805603

[bib45] Tanigaki, K., H. Han, N. Yamamoto, K. Tashiro, M. Ikegawa, K. Kuroda, A. Suzuki, T. Nakano, and T. Honjo. 2002. Notch-RBP-J signaling is involved in cell fate determination of marginal zone B cells. Nat. Immunol. 3:443–450. 10.1038/ni793 11967543

[bib46] Thomas, J.M., S. Surendran, M. Abraham, D. Sasankan, S. Bhaadri, A. Rajavelu, and C.C. Kartha. 2018. Gene expression analysis of nidus of cerebral arteriovenous malformations reveals vascular structures with deficient differentiation and maturation. PLoS One. 13:e0198617. 10.1371/journal.pone.0198617 29897969 PMC5999265

[bib47] Wang, Y., M. Nakayama, M.E. Pitulescu, T.S. Schmidt, M.L. Bochenek, A. Sakakibara, S. Adams, A. Davy, U. Deutsch, U. Lüthi, . 2010. Ephrin-B2 controls VEGF-induced angiogenesis and lymphangiogenesis. Nature. 465:483–486. 10.1038/nature09002 20445537

[bib48] Wooderchak-Donahue, W.L., J. McDonald, B. O’Fallon, P.D. Upton, W. Li, B.L. Roman, S. Young, P. Plant, G.T. Fülöp, C. Langa, . 2013. BMP9 mutations cause a vascular-anomaly syndrome with phenotypic overlap with hereditary hemorrhagic telangiectasia. Am. J. Hum. Genet. 93:530–537. 10.1016/j.ajhg.2013.07.004 23972370 PMC3769931

[bib49] You, L.R., F.J. Lin, C.T. Lee, F.J. DeMayo, M.J. Tsai, and S.Y. Tsai. 2005. Suppression of Notch signalling by the COUP-TFII transcription factor regulates vein identity. Nature. 435:98–104. 10.1038/nature03511 15875024

[bib55] Zhou, Z., A.T. Tang, W.-Y. Wong, S. Bamezai, L.M. Goddard, R. Shenkar, S. Zhou, J. Yang, A.C. Wright, M. Foley, . 2016. Cerebral cavernous malformations arise from endothelial gain of MEKK3–KLF2/4 signalling. Nature. 532:122–126. 10.1038/nature17178 27027284 PMC4864035

[bib50] ZhuGe, Q., M. Zhong, W. Zheng, G.Y. Yang, X. Mao, L. Xie, G. Chen, Y. Chen, M.T. Lawton, W.L. Young, . 2009. Notch-1 signalling is activated in brain arteriovenous malformations in humans. Brain. 132:3231–3241. 10.1093/brain/awp246 19812212 PMC2792368

[bib51] Zuurbier, S.M., and R. Al-Shahi Salman. 2019. Interventions for treating brain arteriovenous malformations in adults. Cochrane Database Syst. Rev. 9:CD003436. 10.1002/14651858.CD003436.pub4 31503327 PMC6735449

